# *Europatitan eastwoodi*, a new sauropod from the lower Cretaceous of Iberia in the initial radiation of somphospondylans in Laurasia

**DOI:** 10.7717/peerj.3409

**Published:** 2017-06-27

**Authors:** Fidel Torcida Fernández-Baldor, José Ignacio Canudo, Pedro Huerta, Miguel Moreno-Azanza, Diego Montero

**Affiliations:** 1 Museo de Dinosaurios, Salas de los Infantes, Burgos, Spain; 2 Colectivo Arqueológico-Paleontológico Salense, Salas de los Infantes, Burgos, Spain; 3 Paleontología, Facultad de Ciencias, Universidad de Zaragoza, Zaragoza, Spain; 4 Grupo Aragosaurus—IUCA, Universidad de Zaragoza, Zaragoza, Spain; 5 Departamento de Geología, Escuela Politécnica Superior de Ávila, Universidad de Salamanca, Salamanca, Spain; 6 GeoBioTec, Departamento de Ciências da Terra, Faculdade de Ciências e Tecnologia, Universidade NOVA de Lisboa, Caparica, Portugal; 7 Museu da Lourinhã, Lourinhã, Portugal

**Keywords:** Dinosauria, Sauropoda, Fossil bones, New species, Early Cretaceous, Spain, *Europatitan eastwoodi*, Titanosauriformes, Somphospondyli

## Abstract

The sauropod of El Oterillo II is a specimen that was excavated from the Castrillo de la Reina Formation (Burgos, Spain), late Barremian–early Aptian, in the 2000s but initially remained undescribed. A tooth and elements of the axial skeleton, and the scapular and pelvic girdle, represent it. It is one of the most complete titanosauriform sauropods from the Early Cretaceous of Europe and presents an opportunity to deepen our understanding of the radiation of this clade in the Early Cretaceous and study the paleobiogeographical relationships of Iberia with Gondwana and with other parts of Laurasia. The late Barremian–early Aptian is the time interval in the Cretaceous with the greatest diversity of sauropod taxa described in Iberia: two titanosauriforms, *Tastavinsaurus* and *Europatitan*; and a rebbachisaurid, *Demandasaurus*. The new sauropod *Europatitan eastwoodi* n. gen. n. sp. presents a series of autapomorphic characters in the presacral vertebrae and scapula that distinguish it from the other sauropods of the Early Cretaceous of Iberia. Our phylogenetic study locates *Europatitan* as the basalmost member of the Somphospondyli, clearly differentiated from other clades such as Brachiosauridae and Titanosauria, and distantly related to the contemporaneous *Tastavinsaurus*. *Europatitan* could be a representative of a Eurogondwanan fauna like *Demandasaurus*, the other sauropod described from the Castrillo de la Reina Formation. The presence of a sauropod fauna with marked Gondwananan affinities in the Aptian of Iberia reinforces the idea of faunal exchanges between this continental masses during the Early Cretaceous. Further specimens and more detailed analysis are needed to elucidate if this Aptian fauna is caused by the presence of previously unnoticed Aptian land bridges, or it represents a relict fauna from an earlier dispersal event.

## Introduction

The vertebrate faunas of the Early Cretaceous of the Iberian Peninsula are of particular interest on account of the special paleobiogeographical location of the Iberian microplate. The Iberian Peninsula is the Laurasian landmass situated closest to Gondwana, and there are obvious relations between certain Iberian and Gondwanan dinosaurs and other clades in the Early Cretaceous, especially in the Barremian (Pereda-Suberbiola et al., 2003; Gheerbrant & Rage, 2006; Canudo, Royo-Torres & Cuenca-Bescós, 2008; Canudo et al., 2009; Torcida Fernández-Baldor et al., 2011; Carballido et al., 2012; Gasca, Canudo & Moreno-Azanza, 2014). One hypothesis that explains how the faunal exchange between Africa and Europe could be developed is the so-called “trans-Tethys” route or Apulian route (Gheerbrant & Rage, 2006). According to this hypothesis, the existence of archipelagos separated by shallow seas of changing eustatic levels, would make possible the migratory movement of the dinosaurs between the two continental masses quoted in a bidirectional sense. The Apulian route could have facilitated these migratory movements intermittently until the Eocene (Gheerbrant & Rage, 2006; Canudo et al., 2009). Bearing this fact in mind, modern paleobiogeographical models point out that Europe and “Gondwanan” territories possessed a common *Eurogondwanan* fauna during the earliest Cretaceous, but that from the Barremian onwards dispersal took place independently in Gondwana and Laurasia, with the isolation of the European faunas (Ezcurra & Agnolín, 2012). To resolve this paleobiogeographical problem, new dinosaur material needs to be put in its correct phylogenetic position and its age established. This may be the only way of ascertaining whether the paleobiogeographical complexity of the Iberian Peninsula in the Early Cretaceous was the result of processes of dinosaur dispersal only at certain points in time or came about as a continuous process. A particularly interesting group for studying this question is the sauropod dinosaurs due to their broad distribution on a worldwide scale in the Cretaceous.

Sauropods are one of the groups of dinosaurs of the Spanish Cretaceous of which our knowledge has increased most substantially as a result of recent discoveries such as those of the macronarians of the end of the Jurassic and the Early Cretaceous. Accordingly, *Galvesaurus* has been described in the Tithonian (Barco et al., 2005; Aurell et al., 2016), *Aragosaurus* at the base of the Cretaceous (Sanz et al., 1987; Canudo et al., 2012; Royo-Torres et al., 2014), *Tastavinsaurus* and *Demandasaurus* in the Barremian–lower Aptian (Canudo, Royo-Torres & Cuenca-Bescós, 2008; Royo-Torres, Alcalá & Cobos, 2012; Torcida Fernández-Baldor et al., 2011), and *Lirainosaurus* and *Lohuecotitan* in the upper Campanian (Sanz et al., 1999; Vila et al., 2012; Díez Díaz et al., 2016). In the Cretaceous, Titanosauriformes were the dominant—indeed almost the only—sauropods in the Iberian Peninsula, as shown by the fact that the vast majority of remains found have been assigned to this clade, with the exception of the rebbachisaurid *Demandasaurus* of the upper Barremian–lower Aptian (Torcida Fernández-Baldor et al., 2011). The systematic position of the macronarian *Aragosaurus*, found at the base of the Cretaceous of Teruel, Spain (Aurell et al., 2016), is a matter of controversy: some authors recover it as a non-titanosauriform macronarian (Mannion et al., 2013; Royo-Torres et al., 2014), whereas for others it possesses characters that suggest its inclusion in Titanosauriformes (Canudo et al., 2012).

Titanosauriformes is the most diverse sauropod clade in the Cretaceous, and is represented on all the continents (D’Emic, 2012; Mannion et al., 2013). More derived titanosauriforms, i.e., lithostrotian titanosaurs, are characterized by apomorphies that have made it possible to identify them relatively easily (Salgado, Coria & Calvo, 1997; Wilson, 2002; González Riga, 2003). However, non-titanosaurian titanosauriforms have been the subject of different interpretations in different cases (Wilson & Upchurch, 2009). This disagreement is due to the scarcity of complete specimens, which has made it difficult to establish synapomorphies that might allow us to distinguish different groups other than the titanosaurs; another difficulty in this sense is the existence of clade definitions that offer different diagnostic characteristics (Salgado, Coria & Calvo, 1997; Wilson, 2002; González Riga, 2003). Titanosauriforms are important to Cretaceous paleobiogeography because of their diversity and ubiquity, but their impact on paleobiogeography has not been fully realized owing to confusion over their phylogenetic *relationships* (D’Emic, 2012; Mannion et al., 2013; Gorscak & O’Connor, 2016; Poropat et al., 2016). Resolving the role of endemism and the details of the faunal turnover of these sauropods depends on ascertaining their lower-level phylogenetic relationships. D’Emic (2012) has undertaken a revision of the Titanosauriformes, proposing a new phylogenetic framework that differentiates two further clades in addition to the titanosaurians: on the one hand the brachiosaurids, with their origin in the Late Jurassic of Pangaea, and on the other hand a second clade of Asian somphospondylans, Euhelopodidae, distributed across the Early-mid Cretaceous of Asia. Ksepka & Norell (2010) also identify several taxa of Asian titanosauriforms as somphospondylans and they point out that there is not evidence of Asian brachiosaurids. The proposal of D’Emic (2012) includes a number of non-titanosaurian Laurasian and Gondwanan genera (*Tastavinsaurus*, *Sauroposeidon*, *Ligabuesaurus*) that are not accommodated within these two clades (see also Mannion et al., 2013). This hypothesis is not the only one that has been proposed, since Royo-Torres, Alcalá & Cobos (2012) have identified a clade they designate Laurasiformes containing Iberian and North American taxa from the Early Cretaceous, bringing together various earlier proposals (Canudo & Cuenca-Bescós, 2004). In spite of these significant contributions to what is known of the phylogenetic relations among the basal titanosauriforms, as well as their paleobiogeographical relationships (especially those relations between Gondwana and Laurasia), further specimens are required to shed new light on the problem.

A particularly prolific area when it comes to continental vertebrate fossil remains from the Early Cretaceous of Spain is the region of Salas de los Infantes (Burgos) in the north of the Iberian Peninsula (Sanz, 1983; Pereda-Suberbiola et al., 2003, 2011; Torcida Fernández-Baldor, 2006; Torcida Fernández-Baldor et al., 2005, 2011; Huerta et al., 2012). On the basis of the discoveries of the last 20 years, a project has been undertaken to bring paleontology to the public attention, its cornerstone being the Dinosaur Museum of Salas de los Infantes, where various dinosaur tracksites have also been prepared as exhibits. In this context, the site of El Oterillo II was found in 2003 and excavated in the following years 2004–2006. The specimen in question is the semi-articulated specimen of a sauropod, from which various remains from the cranial and postcranial skeleton have been recovered. These materials were attributed to Titanosauriformes in a preliminary research on the basis of the morphology of the caudal vertebrae (Torcida Fernández-Baldor et al., 2009). The fossils that constitute the holotype are housed in the Dinosaur Museum of Salas de los Infantes (Burgos). The aim of the present paper is to provide a complete description of the sauropod of El Oterillo II, to ascertain its phylogenetic position in relation to the most recent proposals for Titanosauriformes, and to draw relevant paleobiogeographical conclusions.

## Location and Geological Setting

**Figure 1:**
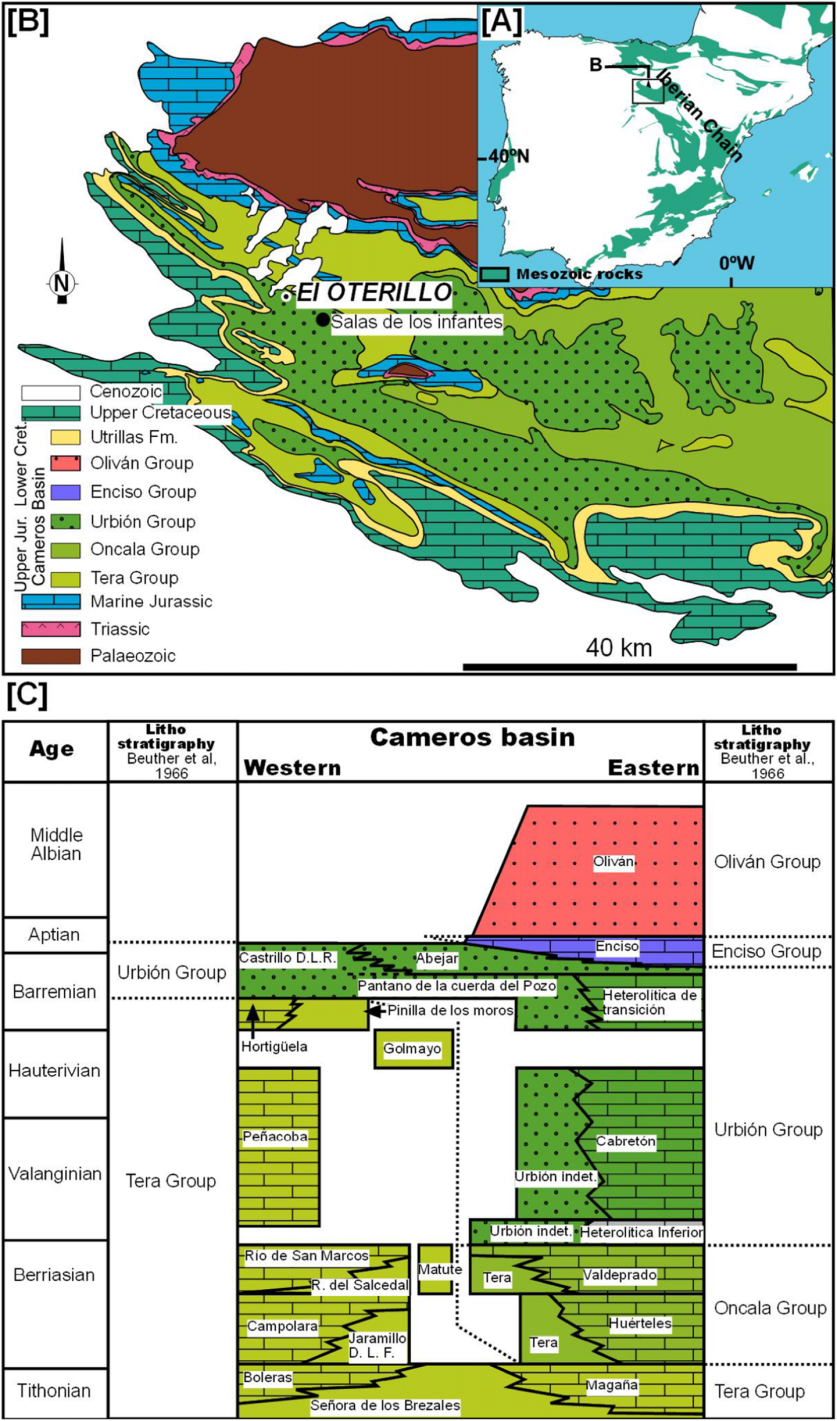
Geological map of the western Cameros Basin. Based on Beuther et al. (1966) indicating the location of El Oterillo site and stratigraphy of the basin modified from Martín-Closas & Alonso Millán (1998).

The site of El Oterillo II is located in the province of Burgos in northern Spain, 2.5 km to the west of the village of Barbadillo del Mercado in Salas de los Infantes (Fig. 1; Fig. S1). Geologically, El Oterillo II is located in the Cameros Basin, which was filled during the upper Jurassic–lower Cretaceous by a non-marine succession (Fig. 1). This basin is a half-graben related to the second phase of the Iberian Rift System, which accumulated around 9,000 m of sediment in its depocenter (Salas & Casas, 1993; Salas et al., 2001). The basin has classically been divided into two sectors. The eastern part is where the depocenter is located and where low-grade metamorphism occurred. It is probably for this reason that fossil bone sites are extremely rare in this area (Canudo et al., 2010), although there are many footprint sites (Castanera et al., 2014). In the western part of the basin (the “*Subcuenca Occidental de Cameros*”), vertebrate bone and track sites are abundant (Torcida Fernández-Baldor, 2006). It is in this part of the basin that the site of El Oterillo II is located. The sediments of the Cameros Basin have traditionally been divided into five groups: Tera, Oncala, Urbión, Enciso, and Olivan. The stratigraphy of the western part of the basin is quite complex due to the different stratigraphic proposals (Platt, 1989; Martín-Closas & Alonso Millán, 1998; Arribas et al., 2003; Clemente Vidal, 2010). The site of El Oterillo II is located in the Urbión Group (Fig. 1).

The bed-to-bed correlation based on aerial photos with stratigraphic logs previously published for the Salas de los Infantes area allows the site of El Oterillo II to be placed in the Castrillo de la Reina Fm., one of the formations included in the Urbión Group (Fig. 1). This lithostratigraphic unit is constituted by alternating sandstone layers ranging from 50 cm to 2 m in thickness with red lutite layers among which there are occasional levels with carbonate encrustations (nodular caliches). These facies are interpreted as distal alluvial plains that record prolonged periods of low clastic sedimentation. As a whole, the sequence represents a distal fluvial–alluvial system originating from the southwest. In the last 10 years, an abundant fossil fauna of dinosaurs and other vertebrates has been described in this formation (Pereda-Suberbiola et al., 2003; Torcida Fernández-Baldor, 2006; Torcida Fernández-Baldor et al., 2005, 2008, 2011, 2015; Pérez-García et al., 2011). The age of the Castrillo de la Reina Fm. is upper Barremian–Aptian, as determined mainly on the basis of charophyte and ostracod biostratigraphy (Martín-Closas & Alonso Millán, 1998; Schudack & Schudack, 2009).

The site of El Oterillo II is located at the top of a sandstone bed with channel geometry, 50 cm thick and 8 m wide, and interbedded with red mudstones. The sandstone bed shows cross-stratification and scattered quartzite clasts. The color of the sandstone is reddish-brown and becomes grey-blue toward the top, where the dinosaur bones appear. In some parts of the site, just below or very close to the bones, a lag of quartzite clasts (1–2.5 cm diameter) with theropod and crocodyliform teeth appears. The paleocurrent measured in the channel indicates an ENE direction. Sandstone channel fills are scarce in the area; other sandstone beds have tabular geometries that are large in extension and centimeters to meters in thickness. The beds dip 15° southwards.

**Figure 2:**
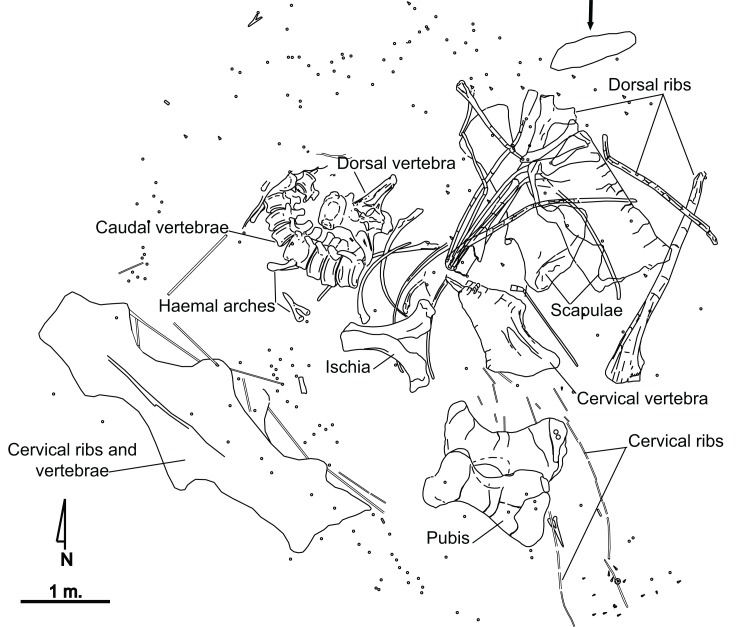
Quarry map of the partial skeleton of *Europatitan eastwoodi* n. gen. n. sp. from the late Barremian–early Aptian, Early Cretaceous, of El Oterillo II site, Spain. The arrow indicates an iguanodontoid ilium (Contreras et al., 2007). Circular symbols correspond to splinters, and triangles to isolated teeth of theropods.

El Oterillo II has yielded the remains of only one sauropod individual (Fig. 2), as well as isolated theropod teeth, which tends to be the case with herbivore carcasses (Alonso, Canudo & Torcida Fernández-Baldor, 2016). An iguanodontian ilium was found at the site although separated from the main bone set (Contreras et al., 2007). A total of 350 fossils belonging to the sauropod specimen have been recovered. A major percentage of these materials are fragments of dorsal and cervical ribs, as well as fragments of vertebral laminae. The most complete material has been studied for the present paper. From the site, a series of caudal vertebrae from the sauropod appears articulated, whereas others such as the hipbones, or the scapula and several ribs, are close to their anatomical position although slightly displaced (Fig. 2). Several bones show tooth marks (Alonso, Canudo & Torcida Fernández-Baldor, 2016), and some are in a poor state of preservation at their distal or proximal ends. Long bones have not been found.

The sandstone bed underlying the dinosaur remains is interpreted as a fluvial channel isolated in floodplain deposits, as is inferred from the geometry, the sedimentary structures, the paleocurrents and the fluvial origin of the Castrillo de la Reina Fm. (Clemente & Perez-Arlucea, 1993; Martín-Closas & Alonso Millán, 1998). The articulation of the sauropod caudal vertebrae and the anatomical position of other bones reveal that transport did not take place. The size of the quartzite clasts and their position below the dinosaur remains suggest that the channel flow was not strong enough to move a sauropod body and that flow occurred before the arrival of the dinosaur. The tooth marks found in some bones, and the presence of several theropod teeth, reveal the presence of carrion feeders. Some parts of the body that are well preserved and do not show tooth marks, such as the caudal vertebrae, were probably covered by water and/or sediment.

## Systematic Paleontology

### Materials and methods

El Oterillo II (OT-II) was found in 2003 during the prospection campaign carried out as part of the Paleontological Inventory of the Sierra de la Demanda (file 243/03-BU JDVR/MCP). The material described in this publication was excavated in 2004, 2005, and 2006 with the corresponding permits from the Heritage Office (*Dirección General de Patrimonio*) of the regional government of Castilla y León (dossiers 307/04-BU; 257/05-BU; 262/06-BU), which is the department responsible for the administration of the paleontological heritage of this region of Spain. Accordingly, the material complies with all the regulations of the Spanish state. All the material described in the present publication is housed in the Dinosaur Museum of Salas de los Infantes (MDS; previously MPS) (Salas de los Infantes, Burgos, Spain), where it is available for comparative study by qualified researchers. The material studied comprises one tooth, five cervical vertebrae, one dorsal vertebra, nine caudal vertebrae, 11 cervical ribs, five dorsal ribs, seven hemal arches, the two scapulae, the left coracoid, the left metacarpals I and III, the two pubes, and the two ischia. The museum numbers are MDS-OTII,1 to MDS-OTII,32.

The electronic version of this article in portable document format will represent a published work according to the International Commission on Zoological Nomenclature (ICZN), and hence the new names contained in the electronic version are effectively published under that Code from the electronic edition alone. This published work and the nomenclatural acts it contains have been registered in ZooBank, the online registration system for the ICZN. The ZooBank LSIDs (life science identifiers) can be resolved and the associated information viewed through any standard web browser by appending the LSID to the prefix http://zoobank.org/. The LSID for this publication is:urn:lsid:zoobank.org:pub:E76E9C58-CB53-4CBE-8CF5-87561A5365A1. The online version of this work is archived and available from the following digital repositories: PeerJ, PubMed Central and CLOCKSS.

### Nomenclature

In general, we use the standardized anatomical nomenclature based on the *Nomina Anatomica Avium* and *Nomina Anatomica Veterinaria* (see Harris, 2004). The nomenclature for the vertebral laminae follows Wilson (1999), with modifications (apcdl) from Salgado, Apesteguía & Heredia (2005) and Wilson et al. (2011). The nomenclature for the vertebral pneumatic structures follows Wedel (2003) and Wilson et al. (2011).

Order SAURISCHIA Seeley, 1887
Infraorder SAUROPODA Marsh, 1878
NEOSAUROPODA Bonaparte, 1986
Titanosauriformes Salgado, Coria & Calvo, 1997
Somphospondyli Wilson & Sereno, 1998
Genus *Europatitan* gen. nov.
urn:lsid:zoobank.org:act:29532C3F-4E3F-4702-845A-2D75EF3C63B
(Figs. 3–17)

**Etymology:** In reference to Europe, the continent where it was found, and the titans, ancient Greek deities known for their gigantic size, endowed with great power.

**Type Species:** *Europatitan eastwoodi* sp. nov., see below.

**Diagnosis:** As for the type and only known species.

*E. eastwoodi* sp. nov.

urn:lsid:zoobank.org:act:B436CCB2-6E5C-498E-80A5-4BF271AC3175.

**Etymology:** Dedicated to US actor Clint Eastwood, the protagonist of the film “The Good, the Bad and the Ugly,” which was partially filmed near Salas de los Infantes.

**Type Locality and Horizon:** The site of El Oterillo II is located in the province of Burgos in northern Spain, 2.5 km to the west of the village of Barbadillo del Mercado in Salas de los Infantes (Fig. 1), Burgos Province, Spain; Urbión Group, Castrillo de la Reina Fm., lower Cretaceous, regarded as late Barremian–early Aptian in age (Martín-Closas & Alonso Millán, 1998).

**Holotype:** MDS-OTII,1 to MDS-OTII-32. The disarticulated carcass of a single specimen consisting of the following material: one tooth, five cervical vertebrae, one dorsal vertebra, nine caudal vertebrae, 11 cervical ribs, five dorsal ribs, seven hemal arches, the two scapulae, the left coracoid, the left metacarpals I and III, the two pubes, and the two ischia.

**Diagnosis:** A large titanosauriform sauropod diagnosed by a combination of autapomorphic and synapomorphic characters. The autapomorphies are as follows: (1) posterior cervical vertebrae with a parapophysis that presents a triradiate laminar structure in its dorsal part dividing the lateral pneumatic fossa; (2) middle and posterior dorsal vertebrae with a horizontal tpol lamina positioned dorsal to the hyposphene; (3) middle and posterior dorsal vertebrae with centroprezygapophyseal lamina joined laterally to two accessory laminae delimiting pneumatic cavities and that partially subdivides the centroprezygapophyseal parapophyseal fossa; (4) in the middle and posterior dorsal vertebrae dorsally the centropostzygapophyseal laminae reach the lateroventral margin of the hyposphene and are forked at their ventral end, (5) middle and posterior dorsal vertebrae with posterior part of the centrodiapophyseal postzygapophyseal fossa broad and divided by various small accessory laminae situated between the posterior centrodiapophyseal and the postzygodiapophyseal laminae, giving rise to small, highly conspicuous pneumatic subfossae; (6) in the middle and posterior dorsal vertebrae there is an accessory lamina present between the anterior and posterior spinodiapophyseal laminae; this lamina divides the fossa situated between the two laminae; (7) on the anterior surface of the capitulum the posterior dorsal ribs present a crest that is sinusoidal in outline running in a proximodistal direction; (8) the dorsal area of the deltoid crest of the scapula presents a sub-elliptical process with a rugose surface, accompanied in its ventral part by a rugose flat area and a pronounced groove; (9) on the dorsal margin of the scapular blade, approximately in its middle part, there is a rugose tubercle with two projections separated by a semicircular depressed area.

The combination of synapomorphic characters is as follows: flat or slightly convex ventral surface of the cervical centra (Ch. 112:0); very reduced pleurocoels in cervical centra (Ch. 114:3) with a well-defined anterior excavation and smooth posterior fossa (Ch. 115:3); dorsal vertebrae with a strongly compressed centrum (Ch. 162:2); procoelous anterior caudal vertebrae (Ch. 193:3); lack of prespinal lamina in the neural arches of the anterior caudal vertebrae (Ch. 207:0); long chevron, hemal canal (Ch. 126:1); rounded expansion on acromial side (Ch. 232:1); well-developed acromion process (Ch. 235:1); ventromedial process of the ventral margin of the scapula well developed (Ch. 237:1); glenoid scapular orientation strongly beveled medially (Ch. 240:1); muscle scar on the proximal end of the ischium (Ch. 291:1); and lack of a ventral bulge on the transverse process of the first caudal (Ch. 358:0).

### Description

**Figure 3:**
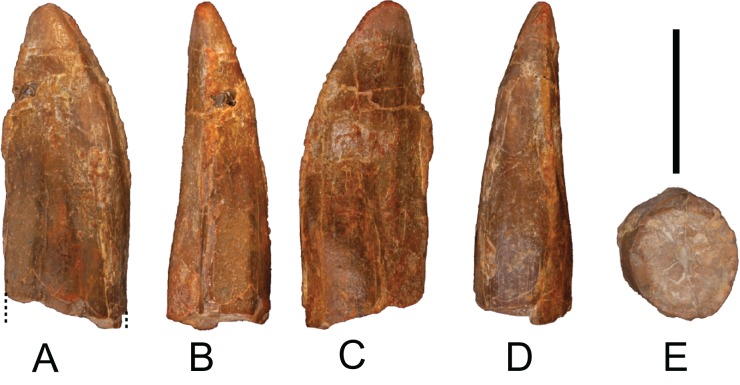
Tooth (MDS-OTII,18) from *Europatitan eastwoodi* n. gen. n. sp. (A) Anterior view. (B) Distal view. (C) Lingual view. (D) Mesial view. (E) Adapical view. Scale: 1 cm.

*Teeth*: One tooth labeled as MDS-OTII,18. This tooth has a complete dental crown, which preserves the base of the pulp cavity and does not have a root (Fig. 3). The overall shape of the tooth is triangular, more spoon-shaped than pencil-shaped, with the crown slightly displaced posteriorly. It is interpreted as being a right maxillary or left mandibular tooth. It is 20 mm in height, 9 mm in mesiodistal width, and its labiolingual width is 4 mm. The approximate value of the slenderness index (SI; Upchurch, 1998) is 2.2. Diplodocoids and titanosaurians have very slender, peg-like teeth with SI values >4.0 and reduced lingual concavities (Upchurch, 1998), indicating that MDS-OTII,18 cannot be referred to either of these clades. However, the SI values are consistent with referral to a basal titanosauriform (Barrett et al., 2002; Chure et al., 2010). Its section is somewhat flattened lateromedially and oval, slightly asymmetrical and more triangular in the apical zone. The mesial and distal edges present fine carinae without denticles. It has subtle ornamentation with crenulations only visible by light microscopy; it possesses gentle crests running in a longitudinal direction, three on the lingual face and four on the labial face. It is a functional tooth, with an apical wear facet. This feature distinguishes it from basal macronarians such as *Camarasaurus* with a V-shaped facet. In basal titanosauriforms such as *Giraffatitan*, teeth with high-angled mesial and distal wear facets but no apical wear have been described, while others display a combination of apical wear and mesial and distal wear (Barrett et al., 2002). Other non-titanosaurian titanosauriforms show sharply inclined wear facets, as occurs in *Ligabuesaurus* (Bonaparte, González Riga & Apesteguía, 2006). The crown base lacks the cingular structure described in the putative euhelopodid of the Early Cretaceous of Spain (Canudo et al., 2002).

**Figure 4:**
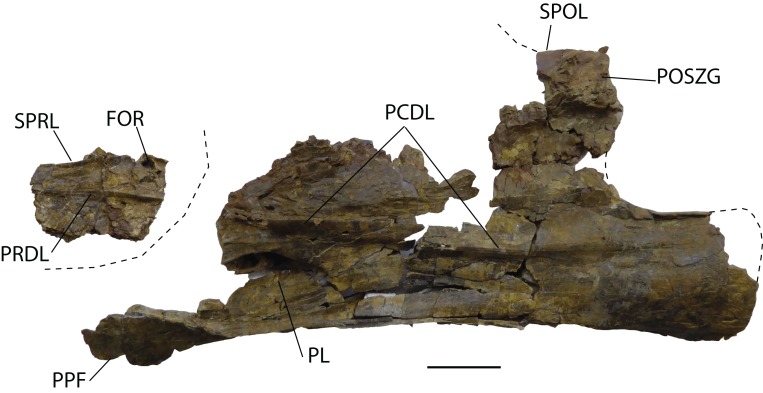
Cervical vertebra (MDS-OTII,32) from *Europatitan eastwoodi* n. gen. n. sp., left lateral view. FOR, foramen; PCDL, posterior centrodiapophyseal lamina; PL, pleurocel; POSZG, postzygapophyses; PPF, parapophyses; PRDL, prezygodiapophyseal lamina; SPOL, spinopostzygapophyseal lamina; SPRL, spinoprezygapophyseal lamina. Scale: 10 cm.

**Figure 5:**
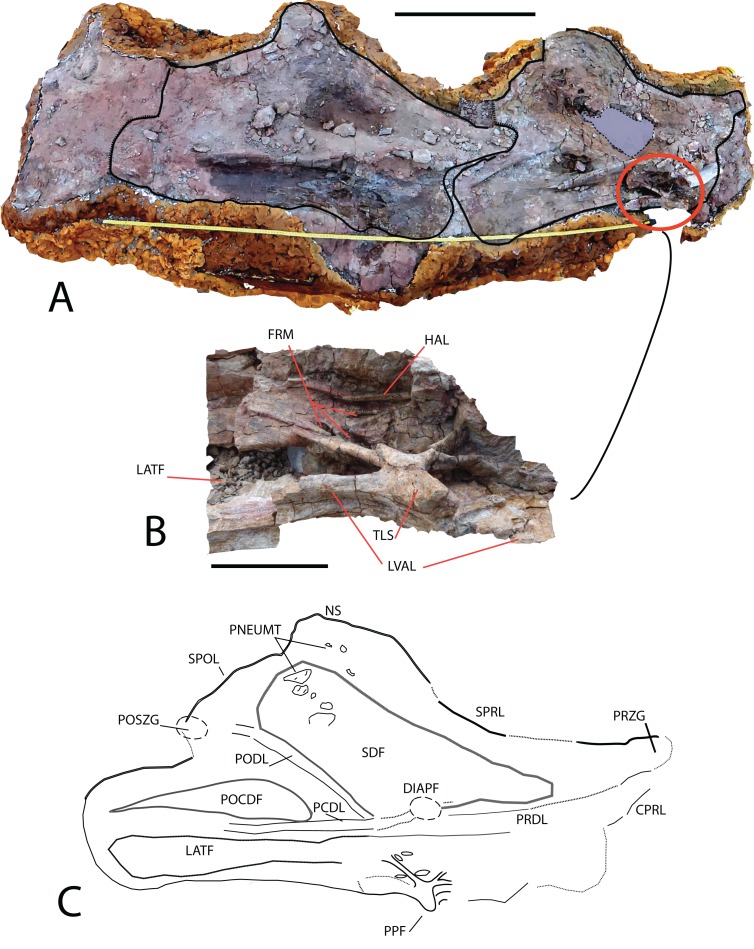
Cervical vertebrae (MDS-OTII,31 A–D) from *Europatitan eastwoodi* n. gen. n. sp. (A) Block from the excavation containing the vertebrae; draw the contour of MDS-OTII,31B and 31C. (B) Detail of triradiated structure in the parapophysis of MS-OTII,31B. (C) Reconstruction of MS-OTII,31B. CPRL, centroprezygapophyseal lamina; DIAPF, diapophysis; FRM, foramina; HAL, horizontal accessory lamina; LATF, lateral fossa; LVAL, lateroventral accessory lamina; NS, neural spine; PCDL, posterior centrodiapophyseal lamina; PNEUMAT, pneumatic subfossae; POCDF, centrodiapophyseal postzygapophyseal fossa; PODL, postzygodiapophyseal lamina; POSZG, postzygapophyses; PPF, parapophyses; PRDL, prezygodiapophyseal lamina; PRZG, prezygapophyses; SDF, spinodiapophyseal fossa; SPOL, spinopostzygapophyseal lamina; SPRL, spinoprezygapophyseal lamina; TLS, trirradiated laminar structure. Scale: 50 cm (A), 10 cm (B).

*Cervical vertebrae*: Five incomplete cervical vertebrae have been recovered. In the cervical series, these could be the 7th (MDS-OTII,32) and the 8th, 9th, 10th, and 11th, which are articulated (MDS-OTII,31A, B, C, D). MDS-OTII,32 preserves the posterior half of the vertebral body, as well as the left postzygapophysis, part of the right parapophysis, and an anterior fragment of the neural arch; its right side is in a very poor condition (Fig. 4). Of the ?A3B2 tlsb?> articulated series, MDS-OTII,31A preserves a small posterior part of the vertebral body; MDS-OTII,31D preserves the most anterior part of the vertebral body, of the neural arch and the neural spine; MDS-OTII,31B and C are almost complete although they have lost some laminae, the diapophysis and part of the parapophysis (Fig. 5). These articulated vertebrae form part of a rocky block in a delicate state of preservation, from which preparation work has made it possible to expose the left side of the vertebrae.

The cervical vertebrae of *Europatitan* are remarkable for their extreme pneumatization and the great anteroposterior lengthening of the vertebral centrum (Table 1), which implies an extremely long neck as displayed by some titanosauriforms, such as *Giraffatitan*, *Sauroposeidon*, and *Erketu* (Janensch, 1950; Wedel, Cifelli & Sanders, 2000a; Ksepka & Norell, 2006).

**Table 1:** Measurements of vertebrae of *Europatitan eastwoodi*.

Vertebra	TW (cm)	TH (cm)	CL (cm)	ACW (cm)	ACH (cm)	PCW (cm)	PCH (cm)	NAH (cm)	NSH (cm)	NSW (cm)	UI	WI
**MDS-OTII,31B**	–	76	114	–	–	–	–	56	44	–	–	–
**MDS-OTII,31C**	–	74	112	–	–	–	–	53	33	–	–	–
**MDS-OTII,32**	–	–	–	–	–	13.5^1^	17^1^	–	–	–	–	–
**MDS-OTII,1**	95	77^1^	24	38	23	43	25	–	–	–	0.56	0.96
**MDS-OTII,2**	45.5	61	14.5	26	31	29	32	32	21	11	0.5	0.45
**MDS-OTII,3**	39.5	57	13.5	2.5	32	26	27	30	8	11	0.52	0.5
**MDS-OTII,4**	35	54	15	26	29	25	27.5	27	7.5	10	0.6	0.54
**MDS-OTII,6**	30.5	44	15.5	25	23	23.5	23	20.5	8	7	0.66	0.67
**MDS-OTII,7**	26	41.5	14.5	24	23	23	22	20	9	6	0.63	0.66
**MDS-OTII,8**	23	37.5	15	22.5	20.5	22	20.5	19	7.5	5.5	0.68	0.73
**MDS-OTII,9**	17	28.5	15.5	17.5	14.5	17	15.5	13.5	8	2.5	0.91	1

**Notes:**

TW, total width; TH, total height; CL, centrum length; ACW, anterior centrum width; ACH, anterior centrum height; PCW, posterior centrum width; PCH, posterior centrum height; NAH, neural arch height; NSH, neural spine height; NSW, neural spine mediolateral width; UI, elongation index *sensu* Upchurch (1998); WI, elongation index *sensu* Wilson (2002). Measurements are in cm.

1 Incomplete or estimate.

MDS-OTII,31B and 31C have a vertebral centrum that is anteroposteriorly lengthened and relatively low, slightly higher than wide (Fig. 4). The vertebral centrum is opisthocoelous with a very marked concavity in its posterior articular face, which is oval. The ventral surface of the centra is transversely concave in its anterior part; in MDS-OTII,32 is, flat with two short and shallow crests in the middle. The lateral surfaces are excavated almost entirely by large pneumatic fossae separated medially by a very fine bony partition. The lateral fossa is perforated by a small pleurocoel that is clearly delimited posteriorly by a sharp edge in MDS-OTII,32 and MDS-OTII,31C. The interior of the pleurocoel is complex, being divided into two parts by laminae that are in turn subdivided by other internal laminae, resulting in a total of six subcavities. Furthermore, there are foramina in each of these subcavities. The pneumatic fossa takes up approximately 80% of the vertebral body, as in *Sauroposeidon* (Wedel, Cifelli & Sanders, 2000a). A horizontal lamina is located dorsal to the pneumatic fossa of the vertebral centrum, delimiting it from the neural arch, like the crest presented by a middle cervical vertebra from the titanosauriform *Astrophocaudia* (D’Emic, 2013), Fig. 5B: HAL.

The parapophysis is located in the anterior half of the vertebral body, which anteriorly and posteriorly has accessory laminae developed on its lateroventral margin (Fig. 5B: LVAL). *Europatitan* shares this characteristic with *Sauroposeidon* and *Giraffatitan* (Janensch, 1929, 1950; Wedel, Cifelli & Sanders, 2000a, 2000b; Rose, 2007). One of these laminae has been described as a posterior centroparapophyseal lamina (pcpl) (Wedel, Cifelli & Sanders, 2000b), but this may not be the homologous lamina described in the dorsal vertebrae, since in the latter the acpl and the pcpl have the neurocentral junction as a landmark, whereas in the cervical vertebrae of *Europatitan* the reference point is the lateroventral margin (Wilson, 1999). In MDS-OTII,31B the parapophysis supports two well-developed accessory laminae in its dorsal part, one of them oriented posteriorly and the other anteriorly. This appears forked at its origin in the wall of the lateral fossa of the vertebral body. Together they form a highly conspicuous triradiate laminar structure whose branches delimit various parts of the lateral pneumatic fossa and contain up to six foramina (Fig. 5B: TLS, FRM). Such a triradiate laminar structure has not been described in other sauropod taxa and is considered an autapomorphy of *E. eastwoodi*.

The neural arch is expanded dorsoventrally and flattened lateromedially. The neural spine is simple, greatly developed dorsoventrally and anteroposteriorly, as a result of which it occupies 80% of the length of the vertebral body. The neural arch presents a subtriangular outline in lateral view. The dorsal margin of the spine has some lateral bumps that are irregular in shape. The lateral surfaces of the neural spine are occupied by a large spinodiapophyseal fossa (sdf), which reaches its greatest depth in its ventral half. In MDS-OTII,31B this fossa presents various minor fossae that are relatively small (8 × 3 cm, 3 × 3.5 cm, 7.5 × 4.5 cm) and have well-delimited margins (Fig. 5C). These fossae are similar to those presented by *Sauroposeidon*, but without the associated development of accessory laminae shown by this taxon (Wedel, Cifelli & Sanders, 2000b). The neural spine also presents various foramina in its anterodorsal and lateral part. You & Li (2009) suggest that the neural spine with deep spinodiapophyseal fossae (sdf) could be a synapomorphy of brachiosaurids, being a character shared by *Giraffatitan*, *Sauroposeidon* and *Qiaowanlong*. However, for other authors *Qiaowanlong* is a more derived taxon, included among the somphospondylans (Ksepka & Norell, 2010; Mannion et al., 2013).

The simple neural spine is a character shared with brachiosaurids, which differentiates it from the Euhelopodidae, which have a bifid neural spine (D’Emic, 2012). It presents a certain simplification regarding the development of bony laminae associated with it. On the anterior surface of the spine there are two well-developed spinoprezygapophyseal laminae (sprl), which delimit the spinoprezygapophyseal fossa (sprf). There is no prespinal lamina (prsl). On the posterior surface, there are two deep spinopostzygapophyseal laminae (spol), which delimit the spinopostzygapophyseal fossa (spof). Spinodiapophyseal laminae (spdl) are absent, as in *Sauroposeidon* (Wedel, Cifelli & Sanders, 2000a, 2000b).

The zygapophyses are not preserved in the vertebral series MDS-OTII,31A, B, C, D, but some observations can be made thanks to the development of the sprl. The prezygapophyses extend beyond the anterior end of the vertebral body, while the postzygapophyses do not reach the posterior limit. The prezygapophyses present deep centroprezygapophyseal laminae (cprl) and laterally they have a prezygodiapophyseal lamina (prdl) in a ventral position. Lateral to the sprl there is a foramen delimited dorsally by a small crest. The left postzygapophysis of MDS-OTII,32 has a subtriangular articular surface and is oriented lateroventrally. Ventral to the postzygapophyses there are centropostzygapophyseal laminae (cpol) and laterally it presents a thick postzygodiapophyseal lamina (podl).

The diapophyses are not preserved, but they can be interpreted as being located in the anterior part of the neural arch, dorsal to the parapophysis. The diapophysis presents prdl and podl, as well as a well-marked posterior centrodiapophyseal lamina (pcdl) with a foramen in its posterior part. The cervical vertebrae of *Europatitan* lack the anterior centrodiapophyseal lamina (acdl), like *Qiaowanlong* (You & Li, 2009). The absence of this lamina is a variable character, for it may be missing in several of the vertebrae of the cervical series (Ksepka & Norell, 2010). This lamina is present in other basal titanosauriforms such as *Giraffatitan* and *Sauroposeidon* (Janensch, 1950; Rose, 2007); in *Giraffatitan* it is a short lamina, and in *Sauroposeidon* it is only found in immature specimens, if *Paluxysaurus* is considered a senior synonym of *Sauroposeidon* (D’Emic, 2013). Finally, between the diapophysis and the podl and pcdl laminae there is a deep postzygapophyseal centrodiapophyseal fossa (pocdf).

**Figure 6:**
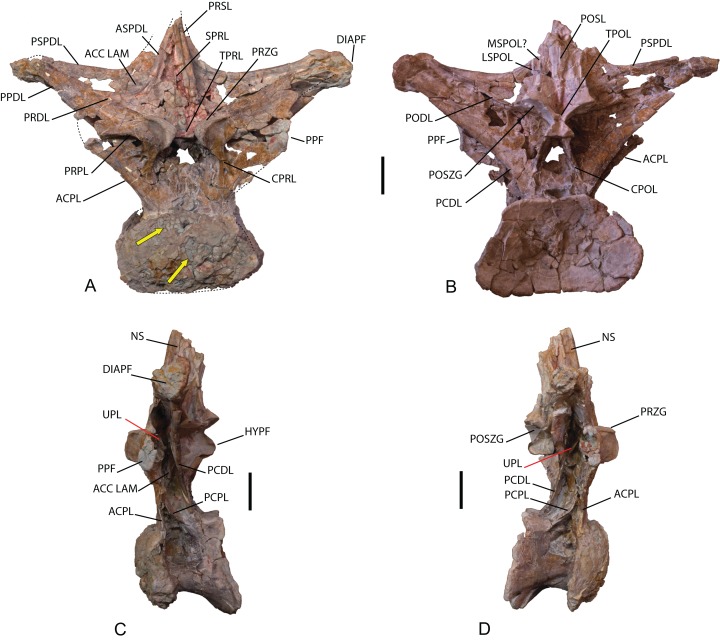
Dorsal vertebra (MDS-OTII,1) from *Europatitan eastwoodi* n. gen. n. sp. (A) Anterior view. (B) Posterior view. (C) Left lateral view. (D) Right lateral view. The arrows in (A) show the pneumatized camellate structure. ACC LAM, accesory lamina; ACPL, anterior centroparapophyseal lamina; ASPDL, anterior spinodiapophyseal lamina; CPOL, centropostzygapophyseal lamina; CPRL, centroprezygapophyseal lamina; DIAPF, diapophysis; HYPF, hyposphenum; LSPOL, lateral spinopostzygapophyseal lamina; MSPOL?, medial spinopostzygapophyseal lamina?; NS, neural spine; PCDL, posterior centrodiapophyseal lamina; PCPL, posterior centroparapophyseal lamina; POSL, postespinal lamina; PODL, postzygodiapophyseal lamina; POSZG, postzygapophyses; PPDL, prezygaparadiapophyseal lamina; PPF, parapophyses; PRDL, prezygodiapophyseal lamina; PRPL, prezygaparapophyseal lamina; PRSL, prespinal lamina; PRZG, prezygapophyses; PSPDL, posterior spinodiapophyseal lamina; SPRL, spinoprezygapophyseal lamina; TPOL, intrapostzigapophyseal lamina; TPRL, intraprezygapophyseal lamina; UPL, unnamed parapophyseal lamina. Scale: 10 cm.

*Dorsal vertebra*: One middle-posterior dorsal vertebra labeled as MDS-OTII,1. Its total height is 77 cm, and its maximum width is 95 cm. It is almost complete and is well preserved; it is only missing the dorsal end of the neural spine and some fragments of the bony laminae (Table 1; Fig. 6; Fig. S2). The vertebral centrum is flattened dorsoventrally, expanded lateromedially, opisthocoelous, wider than long, and approximately as long as it is high, as occurs in macronarians (Wilson & Sereno, 1998; Salgado, Coria & Calvo, 1997). Its articular faces are dorsoventrally compressed, oval in outline, with a greater width in the ventral half; the anterior face is convex, and the posterior concave. The lateral surfaces are anteroposteriorly concave, with a large pleurocoel in the dorsal half, oval in outline and connected to a pneumatic chamber that is deep and well developed both ventrally and anteriorly toward the anterior articular face, and less developed dorsally. Inside the pneumatic chamber there are various scarcely developed laminae. The anterior articular face has lost part of the cortex, making it possible to see its pneumatized internal structure (Fig. 6A), which is of the camellate sort characteristic of Titanosauriformes (Wilson, 2002). The ventral surface is anteroposteriorly concave and smooth, without the medial crest possessed by brachiosaurid titanosauriforms such as *Brachiosaurus* and *Giraffatitan* (Upchurch, Barrett & Dodson, 2004).

**Figure 7:**
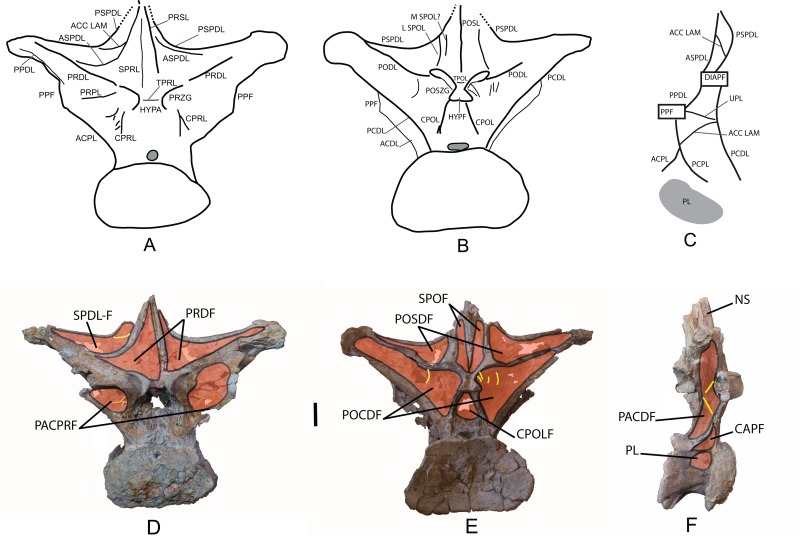
Pneumaticity and lamination of dorsal vertebra MDS-OTII,1 from *Europatitan eastwoodi* n. gen. n. sp. (A–C) Scheme of the laminae. (A) Anterior view. (B) Posterior view. (C) Left lateral view. (D–F) Pneumaticity. (D) Anterior view. (E) Posterior view. (F) Right lateral view. The yellow lines mark the laminae that subdivide the main fossae. ACC LAM, accessory lamina; ACPL, anterior centroparapophyseal lamina; ASPDL, anterior spinodiapophyseal lamina; CAPF, centroparapophyseal fossa; CPOL, centropostzygapophyseal lamina; CPOLF, centropostzygapophyseal fossa; CPRL, centroprezygapophyseal lamina; DIAPF, diapophysis; HYPA, hypantrum; HYPF, hyposphenum; LSPOL, lateral spinopostzygapophyseal lamina; MSPOL?, medial spinopostzygapophyseal lamina?; NS, neural spine; PACDF, centrodiapophyseal parapophyseal fossa; PACPRF, centroprezygapophyseal parapophyseal fossa; PCDL, posterior centrodiapophyseal lamina; PCPL, posterior centroparapophyseal lamina; PL, pleurocelo; POCDF, centrodiapophyseal postzygapophyseal fossa; PODL, postzygodiapophyseal lamina; POSDF, postzygapophyseal spinodiapophyseal fossa; POSL, postespinal lamina; POSZG, postzygapophyses; PPDL, prezygaparadiapophyseal lamina; PPF, parapophyses; PRDL, prezygodiapophyseal lamina; PRPL, prezygaparapophyseal lamina; PRDF, prezygapophyseal spinodiapophyseal fossa; PRSL, prespinal lamina; PRZG, prezygapophyses; PSPDL, posterior spinodiapophyseal lamina; SPDL-F, spinodiapophyseal laminae fossa; SPOF, spinopostzygapophyseal fossa; SPRL, spinoprezygapophyseal lamina; TPOL, intrapostzigapophyseal lamina; TPRL, intraprezygapophyseal lamina; UPL, unnamed parapophyseal lamina. Scale: 10 cm.

**Figure 8:**
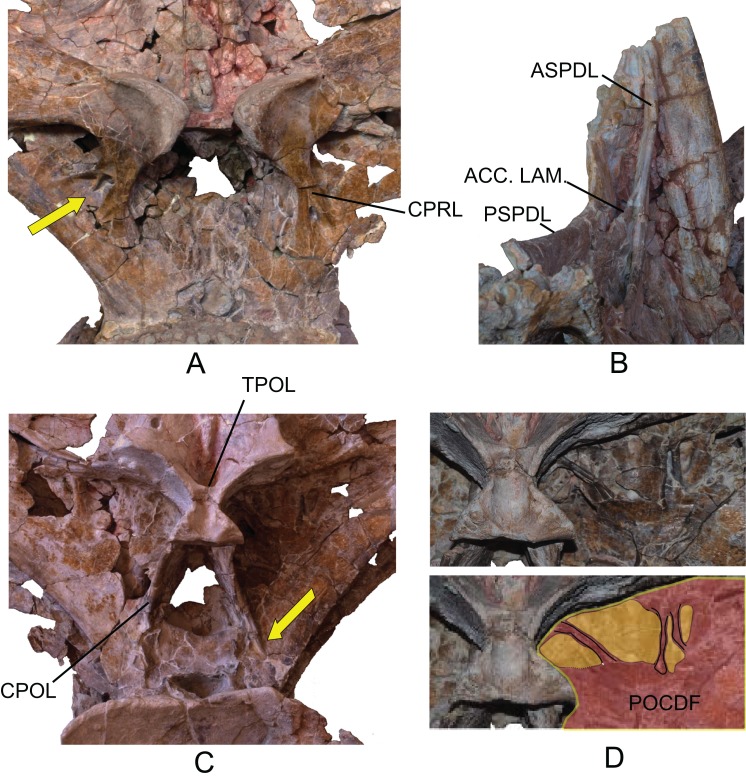
Autapomorphies of *Europatitan eastwoodi* n. gen. n. sp. in the dorsal vertebra MDS-OTII,1. (A) Anterior view, accessory laminae to cprl (arrow). (B) Anterolateral view, accessory lamina between aspdl and pspdl laminae. (C) Posterior view, branched cpol lamina, horizontal tpol lamina. (D) Posterior view (photography and interpretive image), laminae and pneumatic subfossae (yellow colored areas) in the pocdf (red colored area). ASPDL, anterior spinodiapophyseal lamina; CPOL, centropostzygapophyseal lamina; CPRL, centroprezygapophyseal lamina; POCDF, centrodiapophyseal postzygapophyseal fossa; PSPDL, posterior spinodiapophyseal lamina; TPOL, intrapostzigapophyseal lamina.

The neural arch is dorsoventrally elongated, and greatly expanded lateromedially; it is situated in an anterior position on the vertebral centrum. The prezygapophyses are large and thick, reach the anterior margin of the anterior articular face, and are connected to one another by a weak, horizontally developed intraprezygapophyseal lamina (tprl), as occurs in anterior dorsal vertebrae (Figs. 6A and 7A). The presence of this lamina in *Europatitan* is significant in that it tends to disappear with the development of the hyposphene (Wilson, 2002). The articular surface of the prezygapophyses is subrectangular and is slightly inclined ventromedially. Ventrally, the prezygapophyses form a large hypantrum delimited by thick centroprezygapophyseal laminae (cprl). These laminae fork ventrally (Fig. 8A). The right cprl in turn laterally receives two accessory laminae with pneumatic cavities between them, which partially subdivide the centroprezygapophyseal parapophyseal fossa (pacprf). The prezygoparapophyseal laminae (prpl) are horizontal and short. The prezygodiapophyseal lamina (prdl) is a thick, poorly developed ridge that does not reach the diapophysis.

The postzygapophyses are situated at the base of the neural spine and are ventromedially oriented. They are subrectangular and are joined to one another by a short, inconspicuous, horizontal intrapostzygapophyseal lamina (tpol), which is situated dorsal to the hyposphene (Figs. 6B and 7B). The presence of a horizontal tpol lamina in posterior dorsal vertebrae with a hyposphene has been cited in *Sauroposeidon*, if *Paluxysaurus* is considered a senior synonym of *Sauroposeidon* (Rose, 2007; D’Emic, 2013), although other authors do not identify it with this taxon (D’Emic & Foreman, 2012). The development of the hyposphene in middle and posterior dorsal vertebrae tends to be associated with the development of the tpol ventrally to a hyposphene connecting with the neural canal, or with the absence of the tpol (Apesteguía, 2005a; Wilson, 1999).

The hyposphene has a triangular outline in posterior view and a vertical fossa in its central part. Dorsally, the centropostzygapophyseal laminae (cpol) reach the lateroventral margin of the hyposphene and are forked at their ventral end (Fig. 8C). The postzygodiapophyseal lamina (podl) joins the pspdl before it reaches the diapophysis. Ventral to the hyposphene there is a deep centropostzygapophyseal fossa (cpolf). In posterior view the centrodiapophyseal postzygapophyseal fossa (pocdf) is large and is subdivided by various accessory laminae, as many as six on the right side, which are arranged between the pcdl and the podl, giving rise to small but conspicuous pneumatic subfossae. This character is not described in other sauropods (Fig. 8D). In *Sauroposeidon* anterior dorsal vertebrae have been documented with laminae in a similar position to what is described in *Europatitan*, but in the posterior dorsal vertebrae of *Sauroposeidon* there is only one lamina that joins the pcdl and podl laminae, dividing the pocdf fossa into two clearly differentiated subfossae (D’Emic & Foreman, 2012, Figs. 3.2, 4, 6.2).

The neural spine seems to be short. There is a prominent, thick and rugose prespinal lamina (prsl) on its anterior surface, with striations and grooves running dorsoventrally (Fig. 7); the spinoprezygapophyseal laminae (sprl) follow a trajectory parallel to the prsl on the neural spine, until they disappear dorsally, as occurs in *Trigonosaurus* and *Rapetosaurus* and other titanosauriforms (Powell, 1987; Martínez et al., 2004; Campos et al., 2005; Curry Rogers, 2009). There are anterior and posterior spinodiapophyseal laminae (aspdl and pspdl) crossing the lateral surface of the neural spine (Figs. 6 and 7) and delimit an interlaminar fossa that we propose to be named spdl-f, Fig. 7D. These two laminae are present in the dicraeosaurid *Brachytrachelopan* and in titanosaurians (González Riga, 2003; Martínez et al., 2004; Rauhut et al., 2005; Salgado & Coria, 2009; Salgado & Powell, 2010). The right aspdl and pspdl laminae are in contact with one another by means of an accessory lamina that divides the fossa situated between the two spinodiapophyseal laminae (spdl), (Figs. 6C, 7A, 7C and 8B). The posterior surface of the neural spine preserves in its most ventral part a thick, rugose structure that would correspond to a postspinal structure, situated inside a deep spinopostzygapophyseal fossa (spof). The lateral spinopostzygapophyseal lamina (lspol) starts from the postzygapophysis and joins the pspdl to form a compound lateral lamina of the neural spine, as occurs in *Argentinosaurus* and *Epachthosaurus* (Salgado & Powell, 2010). At the base of the neural spine, in an intermediate position between the lspol and the postspinal structure, there are some scarcely developed crests that run toward the lateral lamina or toward the postspinal structure. It is difficult to identify them: they may correspond to medial spinopostzygapophyseal laminae (mspol). There have been citations of lspol laminae in some diplodocimorphs and in Brachiosauridae. Salgado et al. (2004) point out that the lspol join at the neural spine to form a posterior medial lamina (posl).

The diapophyses are oriented almost horizontally. The parapophyses are situated at the height of the prezygapophyses, and join the diapophyses via the paradiapophyseal lamina (ppdl). In lateral view, the posterior centrodiapophyseal lamina (pcdl) is very prominent, and it is wide in posterior view; it runs parallel to the anterior centroparapophyseal lamina (acpl), and between them there is an extensive centrodiapophyseal parapophyseal fossa (pacdf). Two accessory laminae divide the pacdf. One of the laminae is parallel and situated dorsal to the centroparapophyseal lamina (pcpl), between the parapophysis and the pcdl. A similar lamina has been described in *Neuquensaurus* as upl (D’Emic & Foreman, 2012), and it is present in other titanosaurians such as *Rocasaurus*, *Saltasaurus*, *Rapetosaurus*, and *Opisthocoelicaudia* (Salgado, Apesteguía & Heredia, 2005). The other accessory lamina, ventral to the upl and located within the pacdf fossa, runs between the acpl lamina and the pcdl lamina, and is the same lamina as that possessed by the ninth dorsal vertebra of *Neuquensaurus* (Salgado, Apesteguía & Heredia, 2005, Fig. 4C). Ventral to the pacdf fossa is the centroparapophyseal fossa (cpaf), delimited dorsally by the pcpl lamina (posterior centroparapophyseal), which is scarcely developed and joins the acpl in its middle part (Fig. 7F).

**Figure 9:**
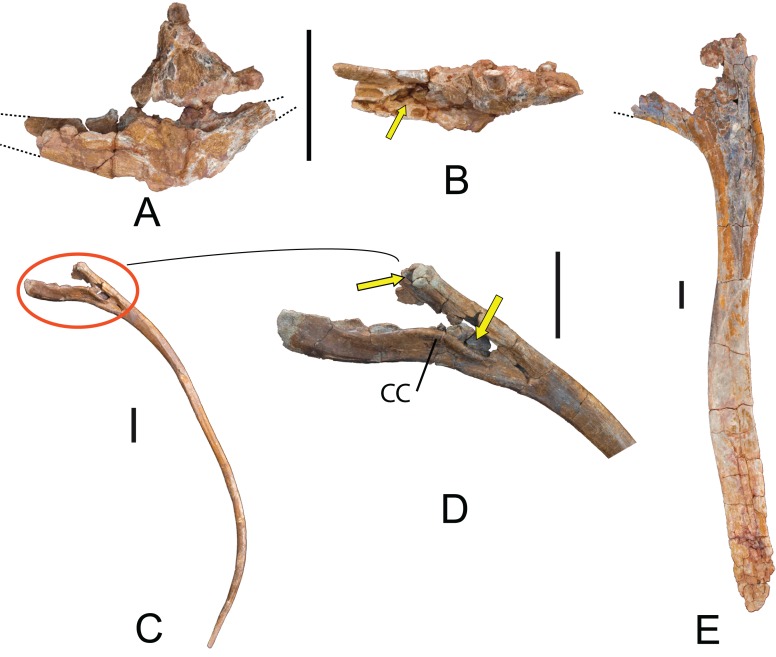
Cervical and dorsal ribs of *Europatitan eastwoodi* n. gen. n. sp. Cervical rib MDS-OTII,24 in (A) medial view and (B) dorsal view; arrow in (B) indicates the pneumatic fossa in the shaft. (C) Dorsal posterior rib MDS-OTII, 20 in (D) posterior view and detail of crested capitulum (cc) and pneumatopores (arrows). (E) Anterior rib (MDS-OTII,21), anterior view. Scale: 10 cm.

Cervical and dorsal ribs: Forty-six ribs (several fragments included) as MDS-OTII,19-24 and MDS-OTII,33-72. Their posterior process represents most of the cervical ribs. MDS-OTII,24 is from a left cervical rib. Between capitulum and tuberculum there is a deep pneumatic fossa that extends through the dorsal part of the posterior process (Figs. 9A and 9B). The posterior process of MDS-OTII,24 is incomplete, reaching a length of 120 cm. Numerous fragments of the posterior process have also been found; these fossils are biconvex or circular in section and elongated, which seems to suggest that the cervical ribs were very long. MDS-OTII,33 is a rib that articulated with the cervical vertebra MDS-OTII,32, and does not preserve its proximal part. It would be greater than 185 cm in length. The articulated series of cervical vertebrae MDS-OTII,31A, B, C, D is associated with various cervical ribs that at a minimum exceed the length of the vertebral centrum to which they are joined, at least partially reaching the following vertebral centrum.

The dorsal ribs MDS-OTII,19 and MDS-OTII,21 are elongated; they are greater than 200 cm in proximodistal length (Fig. 9E). The shaft is subtriangular. Distally it becomes progressively more flattened anteroposteriorly and greatly expanded in a lateromedial direction, such that its overall shape in anterior view is rectangular. MDS-OTII,19 and MDS-OTII, 21 are two anterior ribs, with the capitulum and tuberculum apparently well developed, and the flattened shaft typical of Titanosauriformes (Wilson, 2002). On the articular head the anterior surface is convex and the posterior concave, a character that is found in *Haplocanthosaurus*, *Camarasaurus*, and rebbachisaurids and is considered a synapomorphy of Neosauropoda (Wilson & Sereno, 1998).

MDS-OTII, 22 has its capitulum and tuberculum clearly separated from one another, and the tuberculum is scarcely developed. These characteristics place it in the middle-posterior part of the dorsal series (Upchurch, Barrett & Dodson, 2004). The ribs MDS-OTII,20 and MDS-OTII,23 would be located in the posterior part of the dorsal series. They present intense pneumatization in their proximal part, with a pneumatic depression in the anterior face at the beginning of the shaft, between capitulum and tuberculum. Further, they possess pneumatopores that give access to pneumatic cavities both in the capitulum and the tuberculum (Figs. 9C and 9D). On MDS-OTII,20 there is a crest that is sinusoidal in outline running proximodistally on the anterior surface of the capitulum, delimiting the pneumatic cavity of the capitulum, a character that is considered autapomorphic for *Europatitan*. The pneumaticity of dorsal ribs is a character described for Titanosauriformes that is also shared by diplodocids and rebbachisaurids, although in titanosauriforms the cavities open by means of pleurocoels on the articular head (Gilmore, 1936; Wilson & Sereno, 1998; Lovelace, Hartman & Wahl, 2007; Mannion et al., 2012; Torcida Fernández-Baldor, 2012).

**Figure 10:**
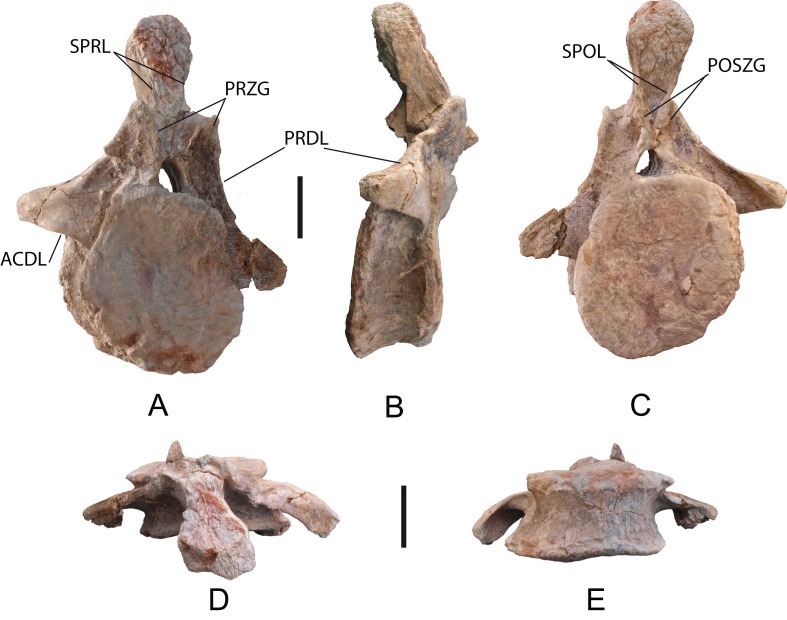
First caudal vertebra (MDS-OTII,2) of *Europatitan eastwoodi* n. gen. n. sp. (A) Anterior view. (B) Right lateral view. (C) Posterior view. (D) Dorsal view. (E) Ventral view. ACDL, anterior centrodiapophyseal lamina; POSZG, postzygapophyses; PRDL, prezygodiapophyseal lamina; PRZG, prezygapophyses; SPOL, spinopostzygapophyseal lamina; SPRL, spinoprezygapophyseal lamina. Scale: 10 cm.

*Caudal vertebrae*: Eight anterior caudal vertebrae labeled as MDS-OTII,2, 3, 4, 5, 6, 7, 8, one middle caudal vertebra (MDS-OTII,9); see Fig. S2. The most anterior vertebra of the caudal series is MDS-OTII,2 (Fig. 10). The vertebral centrum is amphicoelous. The lateral faces are slightly plano-convex dorsoventrally, and concave anteroposteriorly. The ventral surface is concave anteroposteriorly. The neural arch is located in the anterior part of the centrum, as occurs in Titanosauriformes (Salgado, Coria & Calvo, 1997; Wilson, 2002). The transverse processes are laterally projected; they are horizontal and triangular in anterior view (Fig. 10). Between the diapophysis and the vertebral centrum runs the acdl, clearly marked in lateral view. The surfaces of the transverse process present shallow, extensive fossae: in the anterior surface, two centrodiapophyseal prezygapophyseal fossae (prcdf); in the posterior surface, two centrodiapophyseal postzygapophyseal fossae (pocdf). Ventral to the transverse process there is a shallow subcircular fossa. The prezygapophyses are laminar in shape; ventrally they receive the cprl, and dorsally the sprl. The postzygapophyses are reduced; in their dorsal part, they present spol laminae that are very close to one another, delimiting a small but deep spinopostzygapophyseal fossa (spof). There is no hyposphene as presented by derived somphospondylans (Upchurch, 1998; Mannion et al., 2013). The neural spine is posteriorly inclined; its distal end is globular, wide and rugose, with abundant crests and grooves. The section of the spine is subrectangular, and on its lateral faces there are weakly marked spdl laminae, which do not reach the end of the spine.

**Figure 11:**
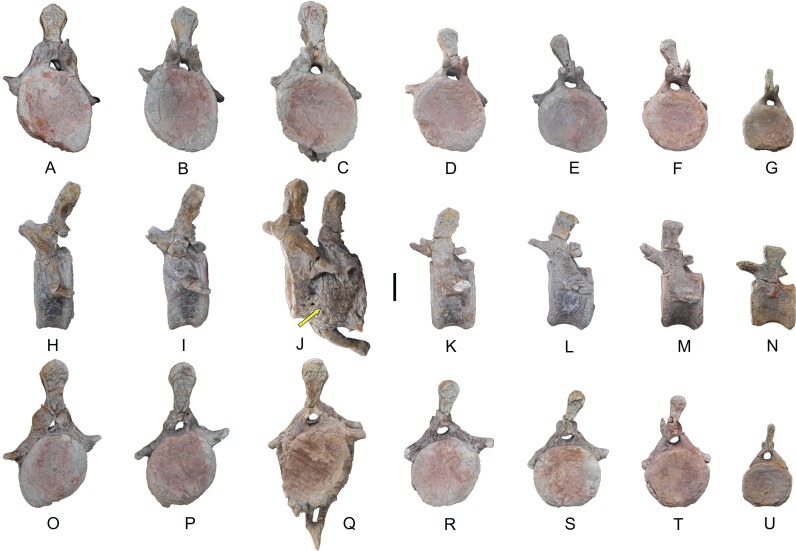
Caudal vertebrae of *Europatitan eastwoodi* n. gen. n. sp. Anterior caudal vertebrae: MDS-OTII,3 (A, H, O), MDS-OTII,4 (B, I, P), MDS-OTII,5 (C, J, Q), MDS-OTII,6 (D, K, R), MDS-OTII,7 (E, L, S), MDS-OTII,8 (F, M, T). Middle caudal vertebra: MDS-OTII,9 (G, N, U), anterior view (A–G), left lateral view (H–N), and posterior view (O–U). The arrow indicates an irregular surface of pathological origin. Scale: 10 cm.

The rest of the anterior caudal vertebrae of *Europatitan* have an anterior articular face that is concave, in some cases deeply so (Fig. 11). The posterior articular face varies from being slightly convex to presenting a concave central part and a convex periphery. This character has been described in Titanosauriformes not included in Titanosauria such as *Venenosaurus* and *Tastavinsaurus* (Tidwell, Carpenter & Meyer, 2001; Canudo, Royo-Torres & Cuenca-Bescós, 2008). Such morphology could represent an incipient procoely in the anterior caudal vertebrae, a primitive state in relation to the procoely of Titanosauria, which show a deep proximal concavity and a highly pronounced distal convexity in the shape of a ball (Salgado, Coria & Calvo, 1997; Canudo, Royo-Torres & Cuenca-Bescós, 2008). Starting from the third caudal vertebra, there appear articular facets for the hemal arches. The neural arch is situated in an anterior position on the vertebral centrum (Figs. 11H–11N). The prcdf, pocdf, and spof fossae decrease in extent and depth until they disappear in the fourth vertebra. The fossae ventral to the transverse processes are very shallow and disappear toward the more posterior vertebrae in the series. The postzygapophyses become increasingly prominent in the course of the series, and in all these vertebrae they receive the cpol laminae. The sprl and spol disappear from the seventh caudal vertebra on. These vertebrae lack a hyposphene, as occurs in Titanosauria (Upchurch, Barrett & Dodson, 2004); this contrasts with the reduced, crest-shaped hyposphene present in some somphospondylans (Mannion et al., 2012; D’Emic, 2012). The neural spines are simple, straight, and posteriorly inclined; they have a club-like dorsal extremity that exhibits conspicuous rugosities on its anterior and posterior faces, like the neural spines described in other macronarians of the Early Cretaceous of Iberia such as *Aragosaurus* and *Tastavinsaurus* (Canudo, Royo-Torres & Cuenca-Bescós, 2008; Royo-Torres et al., 2014).

The middle caudal vertebra of *Europatitan* (MDS-OTII,9) has a spool-shaped centrum that is relatively short and amphicoelous, as in *Tastavinsaurus* and unlike the vertebral centra present in titanosaurians such as *Alamosauru*s and *Saltasaurus* (Powell, Sanz & Buscalioni, 1992; Lehman & Coulson, 2002). The lateral faces of the centrum are concave and smooth; the ventral surface is also concave anteroposteriorly. The neural arch is in an anterior position. It presents reduced sprl laminae, between which a sprf fossa is present. The postzygapophyses are very reduced, presenting spol laminae that delimit a spof fossa. The neural spine is straight, lateromedially compressed, and rugose on its anterior and posterior faces and its dorsal margin (Figs. 11G, 11N and 11U).

**Figure 12:**
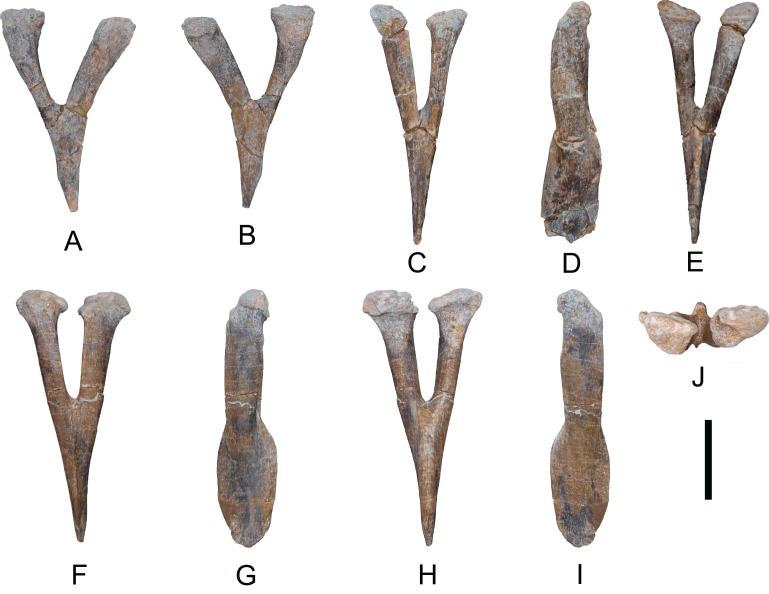
Hemal arches of *Europatitan eastwoodi* n. gen. n. sp. (A) MDS-OTII,27, first hemal arch in anterior view and (B) posterior view. (C) MDS-OTII,25, hemal arch in anterior view, (D) left lateral view, (E) posterior view. (F) MDS-OTII,26, hemal arch in anterior view, (G) left lateral view, (H) posterior view, (I) right lateral view, (J) proximal view. Scale: 10 cm.

*Hemal arches*: Seven hemal arches labeled as 04.17 OT-II,25, 26, 27, 28, 29, and 30; one hemal is fused to MDS-OTII,5 (Fig. 12). The hemal arches are open at the proximal end, a synapomorphy of Neosauropoda (Wilson, 2002). The proximal “crus” bridging the superior margin of the hemal canal is present in some basal sauropods, many flagelicaudatans, and some macronarians, but this character can vary through the caudal series, as in the rebbachisaurid diplodocimorphs (Pereda-Suberbiola et al., 2011; Salgado et al., 2012; Otero et al., 2012). The first hemal arch of the series, MDS-OTII,27 (Figs. 12A and 12B) is articulated with the third and fourth caudal vertebrae, as in *Tastavinsaurus* (Canudo, Royo-Torres & Cuenca-Bescós, 2008). In lateral view the hemal arches are straight, and their proximal end has a double articulation, with a convex anterior surface and a flat posterior surface (Fig. 12J). The surface is smooth and without ornamentation. The first hemal arch is Y-shaped, with dorsal and ventral branches that are similar in length and the ventral branch compressed anteroposteriorly. The separation between the branches is greater than in the rest of the hemal arches (Fig. 12). In all the other hemal arches the ventral branch is lateromedially compressed and is longer than the dorsal branch. The hemal canal (except the first) is roughly 40% of the total length of the hemal arch, similar to *Aragosaurus* and *Tastavinsaurus* (Canudo, Royo-Torres & Cuenca-Bescós, 2008; Royo-Torres et al., 2014); this differentiates them from titanosaurs, which reach values of 50% (Wilson, 2002).

**Figure 13:**
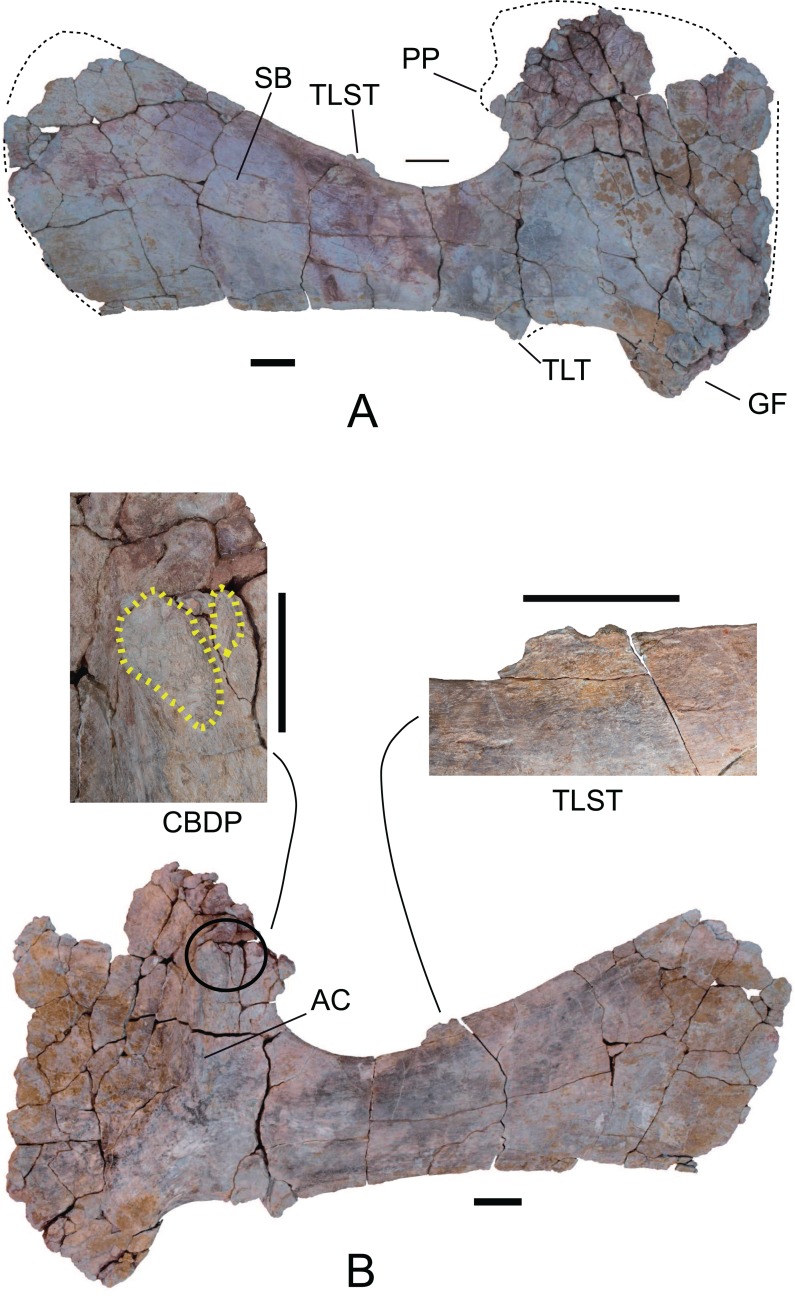
Left scapula (MDS-OTII,14) of *Europatitan eastwoodi* n. gen. n. sp. (A) Medial view. (B) Lateral view with details of two autapomorphic characters detailed in the text. AC, acromial/deltoid crest; CBDP, *coracobrachialis brevis dorsalis* process; GF, glenoid fossa; PP, postacromial process; SB, scapular blade; TLST, *trapezius* and *levator scapulae* tubercle; TLT, *triceps longus* process. Scale: 10 cm.

*Scapulae*: The two scapulae labeled as MDS-OTII,14, left scapula in connection with part of the left coracoid, MDS-OTII,15; MDS-OTII,16, right scapula. The two scapulae are almost complete The left scapula is larger in size than the right one. A description has been made of MDS-OTII,14 (Fig. S3), which has the axis of the scapular blade arranged horizontally. MDS-OTII,14 lacks part of the proximal, proximodorsal and dorsodistal margins of the proximal lamina, as well as part of the distal margin of the scapular blade (Fig. 13). Its general shape is similar to that of Brachiosauridae such as *Giraffatitan* (Janensch, 1950; Curtice, Stadtman & Curtice, 1996) and basal somphospondylans such as *Ligabuesaurus* (Bonaparte, González Riga & Apesteguía, 2006) and *Phuwiangosaurus* (Martin, Suteethorn & Buffetaut, 1999). It differs clearly from the racquet-shaped scapula of Rebbachisauridae (Carballido et al., 2010). The maximum length is 165 cm and as such more than six times the minimum dorsoventral width of the scapular blade, as occurs in many eusauropods and in contrast with basal forms of sauropod such as *Cetiosaurus* (Upchurch & Martin, 2003) and with derived titanosaurians such as *Saltasaurus* (Powell, Sanz & Buscalioni, 1992). The proximal lamina is wide, and the scapular lamina is elongated, with a more pronounced distal expansion on the acromial margin, as shown by certain basal forms of camarasauromorph, where the acromial margin of the scapular blade presents a marked expansion and rounding, as in *Camarasaurus* (Ostrom & McIntosh, 1966) and *Giraffatitan brancai* (Janensch, 1961).

The proximal lamina is up to 150% wider dorsoventrally than the minimum width of the scapular lamina (Harris, 2006) and this ratio reaches a value of 3.5. Values for this character of less than 5.5 are broadly distributed among non-macronarian sauropodomorphs such as *Barapasaurus* (Jain et al., 1979), whereas values greater than 5.5 are seen mainly in the non-titanosaurian macronarians (Borsuk-Bialynicka, 1977; Tidwell, Carpenter & Meyer, 2001; Bonaparte, González Riga & Apesteguía, 2006; Li et al., 2014). Among titanosaurians values below 5.5 are generally observed (Powell, Sanz & Buscalioni, 1992).

In the distal margin of the proximal lamina, part of the postacromial process is preserved (Fig. 13A), which is possessed by various taxa within Titanosauriformes (Bonaparte, González Riga & Apesteguía, 2006; You et al., 2008; Li et al., 2014). The scapulocoracoid articulation ends before the dorsal margin of the acromion, such that the dorsal margin of the coracoid does not reach the dorsal margin of the scapula; D’Emic (2012) includes as a synapomorphy of Saltasauridae a scapulocoracoid suture extends to dorsal margin of acromion and coracoid. The articular face of the glenoid is oriented medially, as in *Apatosaurus* and in somphospondylans (Wilson & Sereno, 1998; Upchurch, Barrett & Dodson, 2004). The acromial or deltoid crest is robust and wide; it forms an angle of 75° with the longitudinal axis of the scapular blade and divides the acromion into two fossae, the anterior of which is wider than the posterior, which has a low lateromedial width. In comparison with other taxa within Macronaria (Harris, 2006, character 207), the dorsalmost point of the acromion is closer to the midpoint of the scapula than to the glenoid.

The dorsal area of the deltoid crest exhibits a sub-elliptical process with a rugose surface, which in its ventral part is accompanied by a rugose flat area and a pronounced groove (Fig. 13B). This process could correspond to the insertion for the *coracobrachialis brevis dorsalis* muscle (Meers, 2003). Two other, very gentle crests with smooth surfaces delimited by grooves lateral to them are present on the deltoid crest in its middle part and would correspond to the insertion for the *scapulohumeralis anterior* muscle (Borsuk-Bialynicka, 1977). Other muscular insertion marks are preserved on the medial surface of the scapular lamina, such as crests and grooves perpendicular to the proximal margin.

The scapular blade expands anterodistally in a uniform manner from its narrowest part toward the distalmost area of the blade. The lateral surface of the scapular blade is convex dorsoventrally, and the medial surface is slightly concave, endowing the scapular blade with a D-shaped cross-section, which becomes weaker distally due to lateromedial flattening. The D-shaped profile is a synapomorphy for the group of *Jobaria* and more derived sauropods, which is present in basal somphospondylans such as *Chubutisaurus* (Carballido et al., 2011). The medial surface of the scapular blade presents a somewhat rugose circular depression in its ventral part where the expansion of the proximal lamina begins, which corresponds to the insertion mark for the *subcoraculoscapularis* muscle in *Opisthocoelicaudia* (Borsuk-Bialynicka, 1977), described as an “eminence” in *Suuwassea* (Harris, 2006).

In the junction between the acromion and the scapular blade there is a triangular process with a rugose surface accompanied by long shallow grooves both on its lateral and medial surfaces (Fig. 13A); these marks would correspond to the insertion for the *triceps longus* muscle (Meers, 2003; Li et al., 2014). It is very prominent, as is the case in the basal somphospondylans *Ligabuesaurus* and *Daxiatitan* (Bonaparte, González Riga & Apesteguía, 2006; You et al., 2008). This process has been described in basal and derived titanosauriforms (Janensch, 1961; Martin, Suteethorn & Buffetaut, 1999; Bonaparte, González Riga & Apesteguía, 2006; Harris, 2007; Carballido et al., 2011; D’Emic, 2012). On the dorsal margin of the scapular blade, approximately in its middle part, there is a rugose tubercle with two projections separated by a semicircular depression (Fig. 13), which could correspond to the insertion for the *trapezius* and *levator scapulae* muscles (Meers, 2003). A similar tubercle has been figured in diplodocoids, where it is a gentle enlargement of the margin of the scapular blade; in basal macronarians such as *Camarasaurus*, where it is very prominent; and in titanosauriforms such as *Giraffatitan* (Janensch, 1950; Curtice, Stadtman & Curtice, 1996; Hocknull et al., 2009). In *Europatitan* the tubercle is divided, which distinguishes it from the rest of the sauropods in which this structure has been cited or figured. Furthermore, its position is more distal in *Europatitan*, except in relation to *Euhelopus* (Young, 1935), where it occupies an intermediate position, similar to *Europatitan*. The insertion marks for the *levator scapulae* muscle extend along the dorsal margin of the medial surface. On the ventral margin of the scapular blade there are insertion marks for the *serratus* muscle, and there are other marks on the distal margin that could correspond to insertions for the *suprascapular* ligament (Borsuk-Bialynicka, 1977; Meers, 2003).

**Figure 14:**
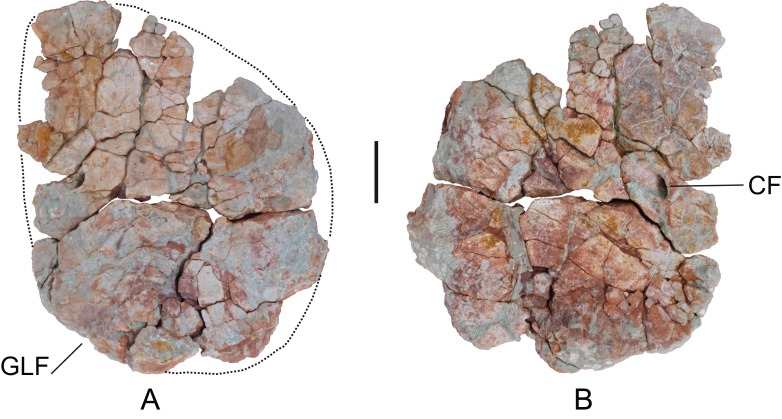
Left coracoid (MDS-OTII,15) of *Europatitan eastwoodi* n. gen. n. sp. (A) Medial view. (B) Lateral view. CF, coracoid foramen; GLF, glenoid fossa. Scale: 10 cm.

*Coracoid*: A left coracoid (MDS-OTII,15), articulated with the left scapula. MDS-OTII,14 is in a poor state of preservation; it is deformed and fractured (Fig. 14). The articulations with the scapula and the dorsoproximal margin are incomplete. It is a quadrangular, equidimensional bone, with a proximodistal length of 52 cm and a dorsoventral length of 59 cm. Its maximum length corresponds to one-third the length of the scapula, and is greater than the scapulocoracoid articulation, a character described in derived titanosaurians (Wilson, 2002; Upchurch, Barrett & Dodson, 2004). The proximal margin is convex and rounded in outline, as occurs in other titanosauriforms such as *Euhelopus*, *Brachiosaurus*, *Paluxysaurus*, *Daxiatitan*, and *Yongjinglong* (Young, 1935; Curtice, Stadtman & Curtice, 1996; Rose, 2007; You et al., 2008; Li et al., 2014). The distal and ventrodistal margins are enlarged, especially the latter, where the enlargement is projected laterally. The coracoid foramen is located close to the distal margin and is closed. The scapulocoracoid articulation forms an angle of roughly 90° with the longitudinal axis of the scapular blade, similar to *Brachiosaurus* (Curtice, Stadtman & Curtice, 1996). An angle of 45° would be a synapomorphy of Nemegtosauridae and more derived titanosaurians (Wilson, 2002), although in a phylogenetic study of *Chubutisaurus* it has also been proposed that it is a synapomorphic character of somphospondylans (Carballido et al., 2011). The medial surface preserves parts with striated areas that correspond to the origin of various muscles: the *triceps longus caudalis* in the glenoid area, and the *supracoracoideus longus* in the dorsodistal area. Likewise, there are marks for the insertion of the *costocoracoideus profundus* muscle near the proximal margin (Meers, 2003).

**Figure 15:**
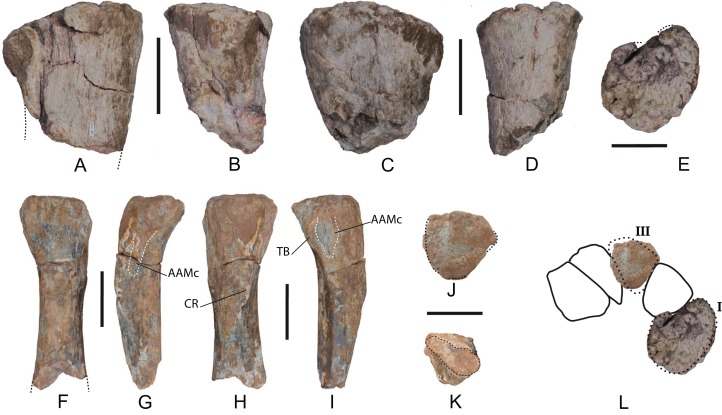
Left metacarpals of *Europatitan eastwoodi* n. gen. n. sp. (1) (A–E) Metacarpal I (MDS-OTII,118). (A) Anterior view. (B) Medial view. (C) Posterior view. (D) Lateral view. (E) Proximal view. (F–K) Metacarpal III (MDS-OTII,17). (F) Anterior view. (G) Medial view. (H) Posterior view. (I) Lateral view. (J) Proximal view. (K) Distal view cross-section of the shaft. (L) Proposed hypothetical reconstruction in proximal view of the set of left metacarpals of *Europatitan*, based on drawing of *Opisthocoelicaudia* (Borsuk-Bialynicka, 1977). AAMc, articular area with metacarpal; CR, crest; TB, tubercle. Scale: 10 cm.

*Metacarpals*: Two proximal fragments of left metacarpals I and III have been recovered (MDS-OTII,118 and MDS-OTII,17, respectively), and part of the diaphysis of metacarpal III (Fig. 15). For the anatomical description it has been taken into account that in proximal view the metacarpals of most sauropods form a semicircle, in such a way that Mc I and V are very close together in the posterior part of the manus (Wilson & Sereno, 1998).

Mc I is robust (Figs. 15A–15E). Its maximum proximodistal length is 20 cm. The proximal articular surface is flat and rugose (Fig. 15E). In proximal view it is oval and D-shaped, being anteroposteriorly expanded (with a width of 19.5 cm); the area of articulation with Mc II is slightly concave. The D-shaped proximal outline of Mc I can be cited in various clades of neosauropods, both primitive and derived, including *Camarasaurus*, *Giraffatitan*, *Aragosaurus*, *Opisthocoelicaudia*, and *Wintonotitan* (Gilmore, 1936; Janensch, 1961; Ostrom & McIntosh, 1966; Borsuk-Bialynicka, 1977; Royo-Torres et al., 2014; Poropat et al., 2015a). The D-shaped proximal outline of MDS-OTII,118 is fairly similar to that of *Opisthocoelicaudia* (Borsuk-Bialynicka, 1977). This characteristic clearly distinguishes it from the compressed morphology presented by other titanosaurians such as *Andesaurus* and *Argyrosaurus* (Apesteguía, 2005b; Mannion & Otero, 2012).

The proximal anterior surface of MDS-OTII,118 is concave, forming a shallow fossa, delimited by two more protruding areas from the medial and lateral surfaces (Fig. 15A). This anterior surface presents grooves and crests that correspond to articulation marks with Mc II and that are prolonged ventrally. More distally, the anterior surface is flat, and it also possesses crests and grooves in its most medial part. The proximal posterior surface is flattened and is slightly convex.

MDS-OTII,17 is the proximal half of the left Mc III (Figs. 15F–15K). Its maximum proximodistal length is 35 cm. In proximal view, it is subtriangular, with its anterior margin wider and its posterior margin narrower. The proximal surface is flat and rugose. It has an anteroposterior width of 12 cm and a lateromedial width of 13 cm. The shaft is twisted in a proximodistal direction and presents one surface oriented anteriorly, another laterally, and another posteromedially, giving rise to a subtriangular cross-section in its proximal part and becoming more oval distally. In anterior view, MDS-OTII,17 is lateromedially wide in the proximal area, becoming thinner distally. Close to the proximal margin there are various short grooves perpendicular to this margin. In lateral view MDS-OTII,17 curves proximally and shows a crest that crosses the surface in a proximodistal direction; it forms a thick, prominent tubercle proximally (Fig. 15I: TB), becoming weaker and sharper distally, as occurs in other titanosauriforms (Apesteguía, 2005b; Hocknull et al., 2009; Poropat et al., 2015a; 2015b). Posterior to the tubercle there is a subtriangular area that corresponds to the articulation with Mc IV (Fig. 15I: AAMc); its surface displays many grooves and crests that give it a rugose appearance. These irregularities develop proximodistally over the whole surface, the development being greater in the proximal half. The medial surface of MDS-OTII,17 also possesses irregularities in its proximal half, which correspond to its articulation with Mc II (Fig. 15G: AAMc). In posterior view, MDS-OTII,17 presents a thick crest in its proximal half (Fig. 15H: CR). The long intermetacarpal articular surfaces shown by *Europatitan* are characteristic of Neosauropoda and are present in diplodocoids, *Camarasaurus* and Titanosauriformes (Wilson & Sereno, 1998) although Apesteguía (2005b) points out that basal titanosauriforms display a reduced dorsal articular contact.

Mc I and Mc III of *Europatitan* differ significantly in their dimensions: the former has an anteroposterior width of 19.5 cm; the latter of 12 cm, 39% less. This ratio is similar to that shown by the metacarpals of *Giraffatitan* and *Wintonotitan* (Janensch, 1961; Poropat et al., 2015a) and almost identical to that of *Opisthocoelicaudia* (Borsuk-Bialynicka, 1977), (Fig. 15L). The relative dimensions of the proximal extremity in other titanosauriforms are variable, and either of the first two metacarpals may be the larger (Bonnan, 2003; Apesteguía, 2005b; (Poropat et al., 2015a; 2015b).

**Figure 16:**
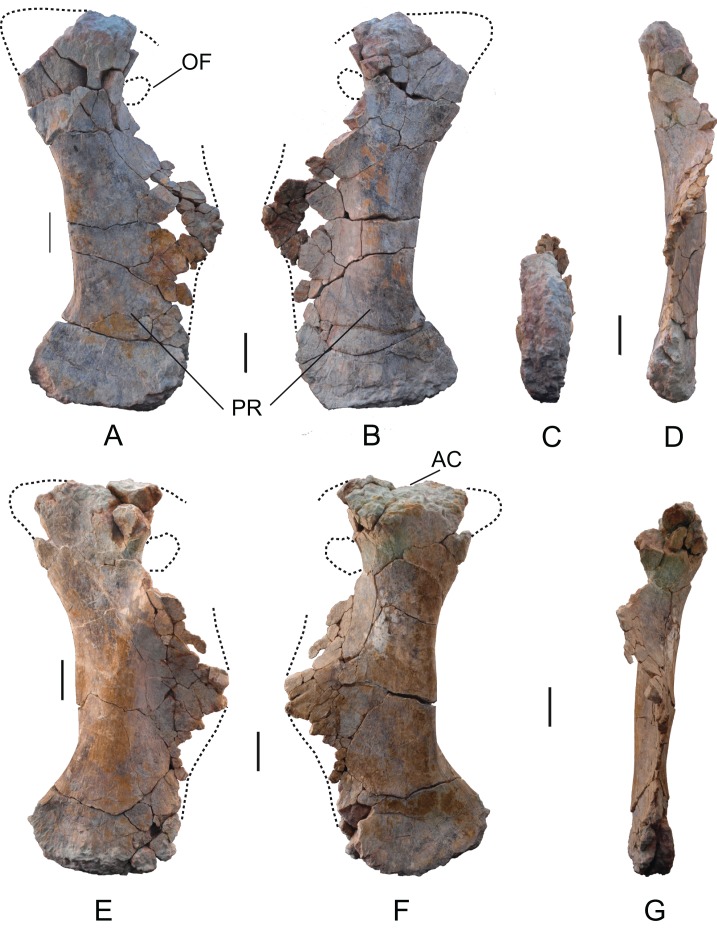
Pubes of *Europatitan eastwoodi* n. gen. n. sp. (A) Right pubis, MDS-OTII,10, in medial view (A), lateral view (B), distal view (C), and posterior view (D). Left pubis, MDS-OTII,11, in lateral view (E), medial view (F), and posterior view (G). AC, acetabulum; OF, obtutator foramen; PR, pubic ramus. Scale: 10 cm.

*Pubis*: Two pubes labeled as MDS-OTII,10 (rigth) and MDS-OTII,11 (left). The left pubis is more complete, although in both cases the anteroproximal corner of the iliac peduncle and a large part of the ischial peduncle are missing, as is the whole of the obturator foramen (Fig. 16). The pubes have a dorsoventral length of 104 cm. They are longer than the ischia (83 cm, 1.3 times longer, Table 2), as in Titanosauriformes (Upchurch, 1998; Calvo & Salgado, 1995); this ratio reaches its highest level in titanosaurids such as *Opisthocoelicaudia* and *Rapetosaurus* (Curry Rogers & Forster, 2001). The relative proportions of ischium and pubis in *Europatitan* are very similar to those obtained for the euhelopodid *Tangvayosaurus* (Allain et al., 1999). The pubes have a robust overall appearance, like the pubis of *Camarasaurus* (Ostrom & McIntosh, 1966), *Giraffatitan* (Janensch, 1961), and somphospondylans (Martin, Suteethorn & Buffetaut, 1999; Salgado & Azpilicueta, 2000; Canudo, Royo-Torres & Cuenca-Bescós, 2008; D’Emic et al., 2013). In posterior view, they are sinuous. The area of the acetabulum is large, and it is slightly concave anteroposteriorly. The lateral surface of this extremity has an area that is slightly concave in its middle part, unlike the medial surface, which is very convex. The obturator foramen is situated ventral to the acetabulum, in the proximal part of the ischial peduncle.

**Table 2:** Measurements of pubes of *Europatitan eastwoodi*.

Pubis	MDS-OTII,10 (Left)	MDS-OTII,11 (Right)
**PL (cm)**	104.5	104
**DEIP (cm)**	70	62^1^
**APW (cm)**	42	40^1^
**IPL (cm)**	45–50^1^	45–50^1^
**APWIP (cm)**	–	38
**MWPS (cm)**	27	28
**MWDE (cm)**	42	40^1^

**Notes:**

PL, proximodistal length; DEIP, distance from distal end to base of ischial peduncle; APW, anteroposterior width (measured at the base of ischial peduncle); IPL, ischial peduncle length; APWIP, anteroposterior width of ischial peduncle; MWPS, anteroposterior minimum width of pubic shaft; MWDE, anteroposterior maximum width distal end. Measurements are in cm.

1 Incomplete or estimate.

The ischial peduncle is well expanded posteriorly and proximodistally. Its lateral surface is convex, and its medial surface concave. Its estimated expansion for *Europatitan* is 0.4, similar to that of basal titanosauriforms and titanosaurians, where the greatest expansion is attained (Royo-Torres, 2009, character C136). *Europatitan* presents a greater expansion of the ischial peduncle than basal eusauropods and diplodocoids. The length of the ischial ramus of the pubis of *Europatitan* is relatively long and similar to that of other primitive titanosauriforms (Royo-Torres, 2009), by contrast with titanosaurids, which present a short ischial ramus (Salgado, Coria & Calvo, 1997). The ratio between the length of the ischial articular surface and the total length of the pubes of *Europatitan* shows values between 0.43 and 0.48, values similar to the ratios displayed by *Giraffatitan* and *Andesaurus* (Ostrom & McIntosh, 1966; Calvo & Bonaparte, 1991). Derived titanosaurids have lower values, i.e., they have a short ischial symphysis (Mannion et al., 2013). *Tastavinsaurus* also displays lower values of around 0.37, the same as *Aragosaurus* (Canudo, Royo-Torres & Cuenca-Bescós, 2008; Royo-Torres, Alcalá & Cobos, 2012; Royo-Torres et al., 2014). The symphysis extends proximally to leave a series of grooves close to the anterior margin of the ischial ramus, occupying almost the whole of it. This character is considered derived and appears in other titanosauriforms (Upchurch, 1998; Wilson, 2002).

The pubic ramus is robust, well-expanded lateromedially and compressed anteroposteriorly, except at its distal end, where it becomes thicker (Fig. 16). The lateral side of the ramus is convex and the medial side straight. The ratio between the anteroposterior width of the ramus in its narrowest part and its distal extremity is 0.48, which indicates a scarcely expanded distal end. This value varies among Titanosauriformes, with values similar to *Europatitan* in *Brachiosaurus;* in the titanosaurians reaches higher values (i.e., relatively low distal expansion) and a weak distal expansion of the pubic ramus. *Tastavinsaurus* is an exception, with a value of 0.36, which corresponds to a distal extremity that is well expanded anteroposteriorly (Canudo, Royo-Torres & Cuenca-Bescós, 2008).

The outline of the distal end in lateral view is slightly convex, with the anterior and posterior margins rounded, the anterior margin somewhat more protruding. This feature distinguishes it from the pubis of *Camarasaurus*, *Aragosaurus*, and *Tastavinsaurus*, which have a very marked projection (Ostrom & McIntosh, 1966; Canudo, Royo-Torres & Cuenca-Bescós, 2008; Royo-Torres, Alcalá & Cobos, 2012; Royo-Torres et al., 2014). In the posteromedial part of the distal end there is a somewhat irregular expansion. The distal articular surface has an ellipsoidal outline, with its major axis running in an anteroposterior direction; it is convex, and in the course of it there emerges a crest.

**Figure 17:**
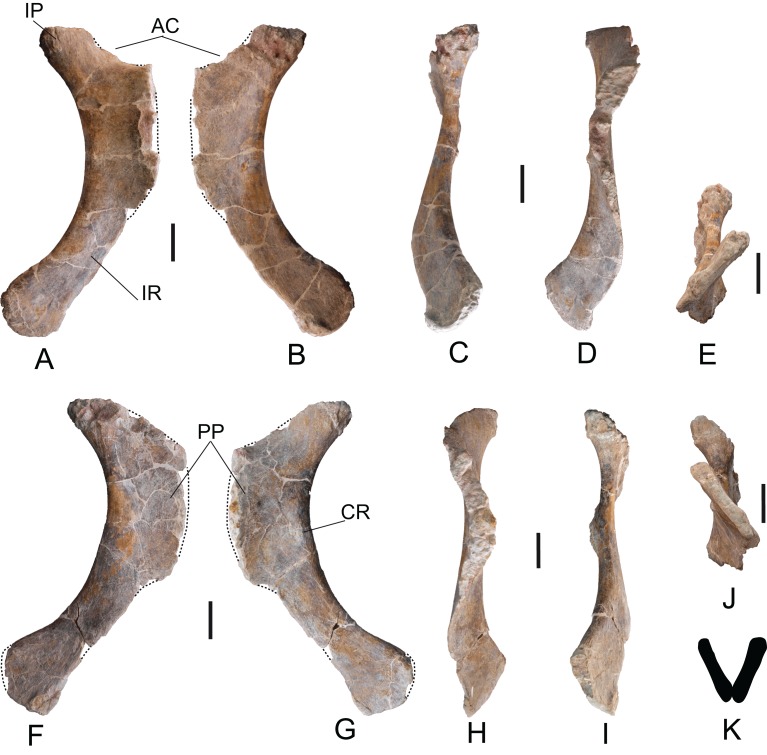
Ischia of *Europatitan eastwoodi* n. gen. n. sp. (A–E) Right ischium, MDS-OTII,12. (F–J) Left ischium, MDS-OTII,13. (A, G) Lateral view. (B, F) Medial view. (C, I) Posterior view. (D, H) Anterior view. (E, J) Posteroventral view. (K) Distal profile of ischia in posterior view. AC, acetabulum; CR, crest; IP, iliac peduncle; IR, ischial ramus; PP, pubis peduncle. Scale: 10 cm.

*Ischium*: Two ischia labeled MDS-OTII,12 (right) and MDS-OTII,13 (left). The ischia are reasonably complete, lacking part of the pubic peduncle (Fig. 17). The ischium of *Europatitan* is smaller in size than the pubis: its dorsoventral length is 83 cm (Table 3). The iliac ramus is clearly differentiated, projecting posteriorly. It has a conic outline in posterior view, with the proximal part the widest. In posterior view, it is rugose, with crests and grooves and many nutrition foramina throughout the proximal part. The lateral surface of the iliac ramus has a crest close to the posterior margin (Fig. 17G: CR), which is associated with a gentle depression in its posterior part corresponding to the insertion for the *flexor tibialis internus* muscle (Borsuk-Bialynicka, 1977). A similar crest has been documented in *Haplocanthosaurus* (Hatcher, 1903) and in rebbachisaurids such as *Demandasaurus* and *Zapalasaurus* (Salgado, García & Daza, 2006; Torcida Fernández-Baldor, 2012). It has also been described in *Aragosaurus* (Sanz et al., 1987), in the somphospondylans *Huabeisaurus* and *Wintonotitan* (D’Emic et al., 2013; Poropat et al., 2015a) and in titanosaurians (Curry Rogers, 2009; Otero, 2010; D’Emic et al., 2013; Gallina & Apesteguía, 2015). The acetabulum has a slightly concave outline, with a very narrow margin in proximal view; it continuously links the iliac and pubic peduncles. Its lateral surface is concave. The contribution of the ischium to the acetabulum is notably, as occurs in most sauropods except *Giraffatitan* and *Tastavinsaurus* (Wilson, 2002; Canudo, Royo-Torres & Cuenca-Bescós, 2008). The pubic ramus in its anatomical position is arranged vertically; it is lateromedially compressed, developed in an anterior direction, and expanded dorsoventrally. Its medial surface is concave, and its lateral surface is convex. Close to the anterior margin, the lateral surface is rugose, with abundant crests and grooves. In anterior view, the pubic symphysis is rugose, thicker in its proximal part and becoming thinner distally.

**Table 3:** Measurements of ischia of *Europatitan eastwoodi*.

Ischium	MDS-OTII,12 (Right)	MDS-OTII,13 (Left)
**PL (cm)**	83	83
**PPL (cm)**	41^1^	37^1^
**APWIP (cm)**	15	–
**MWIS (cm)**	15	14
**MWDE (cm)**	20	21

**Notes:**

PL, proximodistal length; PPL, pubic peduncle length; APWIP, anteroposterior width of iliac peduncle; MWIS, anteroposterior minimum width of ischial shaft; MWDE, anteroposterior maximum width distal end. Measurements are in cm.

1 Incomplete or estimate.

The ischial ramus of *Europatitan* is long. It is lateromedially compressed and progressively expands anteroposteriorly as it develops proximodistally, which gives rise to a rectangular shape in medial view; the ramus expands anteroposteriorly (or dorsoventrally in anatomical position) at the distal end, with a greater expansion in its posterior part, creating a convex margin and as a whole resulting in a distal end with a semicircular outline. The ischial ramus is directed posteroventrally and forms an angle of 48°–50° with the horizontal, a value that falls within the range of variability observed for most sauropods (Royo-Torres, 2009) except *Camarasaurus* and *Lourinhasaurus*, which have a rather horizontal ramus (Ostrom & McIntosh, 1966; Dantas et al., 1998). The distal end has the same lateromedial width as the rest of the ischial ramus. The anterior margin is sharp, and the posterior margin rounded and wider. The ischial symphysis lies in the distal part of the medial surface of the ischial ramus, at its anteroventral end; it is small, rugose, and level in the left ischium, projecting in the right one. The symphysis extends proximally to leave a series of grooves close to the anterior margin of the ischial ramus, occupying almost all of it, as is seen in MDS-OTII,12. An ischial symphyseal joint that extends beyond the distal extremity of the ischial ramus is a derived character present convergently in *Apatosaurus* and in Titanosauria such as *Alamosaurus* and *Opisthocoelicaudia* (Gilmore, 1936; Borsuk-Bialynicka, 1977), where the ischia join together proximally. *Europatitan* possesses ischia that are only fused at their distal end, a primitive character shared with basal macronarians such as *Camarasaurus* (McIntosh et al., 1996) and somphospondylans such as *Tangvayosaurus* and *Tastavinsaurus* (Royo-Torres, 2009; Royo-Torres, Alcalá & Cobos, 2012; D’Emic, 2012). On the posterior margin of the distal part of the ischial ramus there are various grooves and crests that extend very close to the distal end in a very irregular surface; these marks could correspond to the cartilaginous covering of this part of the bone (Borsuk-Bialynicka, 1977).

## Phylogenetic Analysis

To assess the phylogenetic position of *Europatitan* within Eusauropoda, we coded this new taxon in the matrix published by Carballido et al. (2015), a dataset focused on studying the relations among titanosauriforms. The resulting dataset included 75 terminal taxa coded for 370 characters, 20 of which were treated as ordered (12, 21, 58, 95, 96, 106, 108, 115, 116, 120, 145, 152, 163, 213, 216, 232, 233, 234, 252, 256, 299, and 301), and all of them were equally weighted. The resulting matrix (see Data S1 for *Europatitan* codings) was analyzed with TNT 1.1 (Goloboff, Farris & Nixon, 2008). The most-parsimonious trees were sought using a heuristic search, with Wagner starting trees and 1,000 random addition sequences, saving up to 10 trees per replication. Bremer support and bootstrap values after 1,000 replicates were calculated for each branch to assess its robustness. To test the hypothesis of the monophyly of the Spanish stem Somphospondyli, constrained analyses were carried out using TNT (see Data S2 for the exact constrains applied). The resulting trees were subjected to Templeton’s test (Templeton, 1983) using the TNT script by Schmidt-Lebuhn (original script can be downloaded at http://www.anbg.gov.au/cpbr/tools/templetontest.tnt).

**Figure 18:**
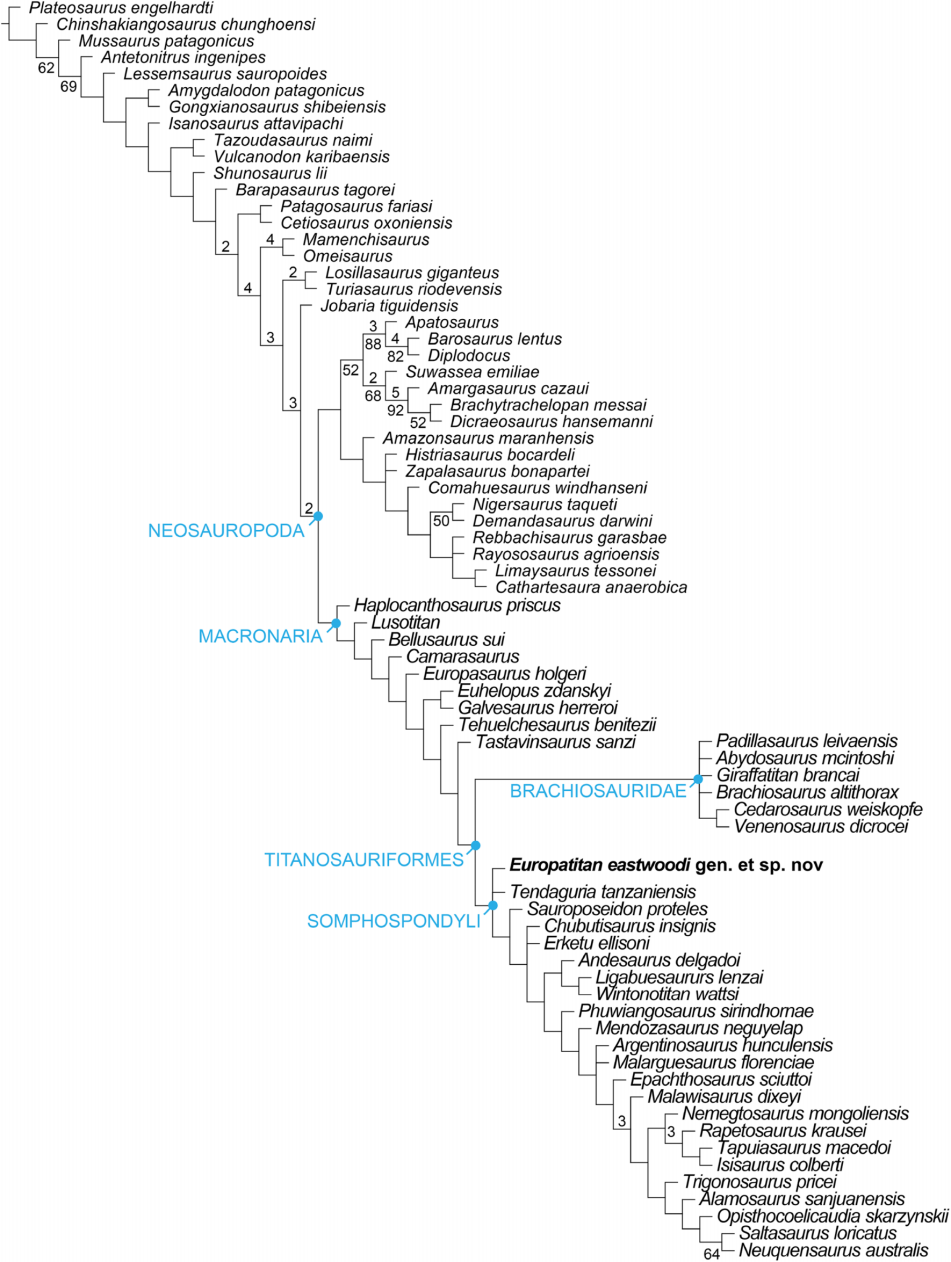
Strict consensus tree showing the phylogenetic relations of *Europatitan eastwoodi* gen. et. sp. nov. within Sauropoda using the matrix of Carballido et al. (2015) (See Appendix 4 for *Europatitan* scores). Strict consensus of five most-parsimonious trees of 1,101 steps. *Europatitan* is recovered as a basal somphospondylan, and more derived than the contemporaneous *Tastavinsaurus*, which is recovered as a non-titanosauriform camarasauromorph. Numbers over nodes represent Bremer support values over 2. Numbers below nodes represent bootstrap values over 50. The topology is better resolved than in previous analyses, but the general support is still low.

Five most-parsimonious trees of 1,101 steps (consistency index = 0.40, retention index = 0.72, rescaled consistency index = 0.29) were recovered (Fig. 18) in 114 of the 1,000 replicates. Further searches using tree bisection reconnection on the existing trees failed to find new most-parsimonious trees. The overall topology of the consensus tree is similar to the tree published by Carballido et al. (2015). *Europatitan* is recovered as a basal member of Somphospondyli (Fig. 18), as it fulfils the definition provided by Wilson & Sereno (1998): “Neosauropods more closely related to *Saltasaurus loricatus* than to *Brachiosaurus altithorax.*” *Europatitan* is recovered in a trichotomy with *Tendaguria* and the clade formed by *Sauroposeidon* and all its descendants. Nevertheless, it is worth noting that, although better resolved, the support for these topologies is as low as in previous analyses. The monophyly of Somphospondyli is supported by a single synapomorphy: the lack of a middle single fossa projected through the midline of the neural spine of the dorsal vertebrae (ch. 144, 0 → 1).

The high diversity of sauropod dinosaurs during the Late Jurassic and Early Cretaceous in Iberia peninsula raises the non-trivial question of the existence of an endemic Iberian clade of Titanosauriformes sauropods. The existence of this clade, either restricted to Iberia or with a slightly wider distribution has been postulated in the past. Royo-Torres (2009) and Royo-Torres, Alcalá & Cobos (2012) recovered a clade of mainly European forms, which they named Laurasiformes. This clade originally included *Aragosaurus, Galvesaurus, Phuwiangosaurus, Venenosaurus, Cedarosaurus, Tehuelchesaurus, Sonorasaurus*, and *Tastavinsaurus*, although its composition and position within Macronaria has varied (Barco, 2010; Carballido et al., 2011) and most recent analysis failed to recover this clade as such (D’Emic, 2012; Upchurch, Mannion & Taylor, 2015; Mocho, Royo-Torres & Ortega, 2016).

To test the existence of this clade, a second version of the dataset was built, this time including the late Berriasian Spanish sauropod *Aragosaurus*. This taxon was coded based in a combination of direct observations of the holotype by one of us (JIC), with the addition of new data based in the new material reported by Royo-Torres et al. (2014). At the time of this study we were not able to perform direct observations on the newly reported material, with this the reason for which we did not include this taxon in our first analysis.

**Figure 19:**
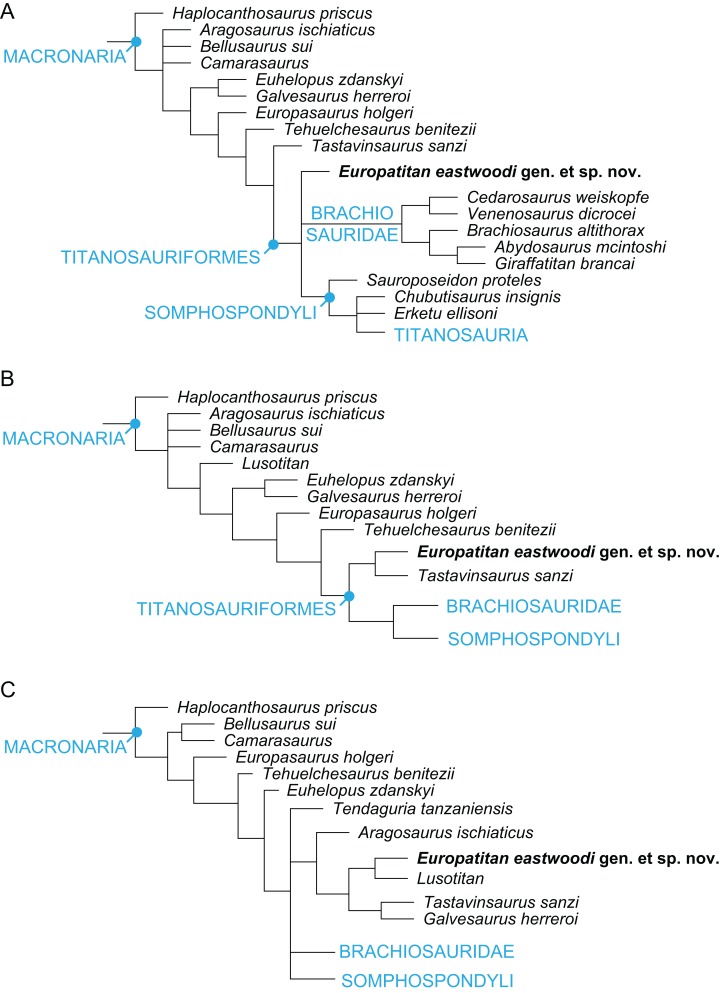
(A) Reduced strict consensus (RSC) tree showing the phylogenetic relations of *Europatitan eastwoodi* within Sauropoda using the matrix of Carballido et al. (2014), with a deletion of *Lusotitan, Tendaguria, and Padillasaurus.* Here, *Europatitan* is recovered in a polytomy with Brachiosauridae and Somphospondyli. (B) First additional constrained analysis enforcing the monophyly of *Tastavinsaurus* + *Europatitan*. The strict consensus places the *Tastavinsaurus* + *Europatitan* clade as the sister taxa of Titanosauriformes (Brachiosauridae + Somphospondyli). (C) Second additional constrained search enforcing the monophyly of *Lusotitan* + *Europatitan*. The strict consensus is poorly resolved, with the Iberian clade recovered in a polytomy with *Tendaguria*, Brachiosauridae and Somphospondyli.

The inclusion of *Aragosaurus* resulted in a total of 76 MPTs of 1,112 steps. The strict consensus is poorly resolved, with the collapse of Brachiosauridae at the base of Macronaria. *Europatitan* is recovered in this polytomy. A posteriori deletion of *Lusotitan, Tendaguria*, and *Padillasaurus*, identified as wildcard taxa with the pruned trees option of TNT, resulted in a better-resolved reduced strict consensus. Here, *Europatitan* is recovered in a polytomy with Brachiosauridae and Somphospondyli (Fig. 19A). Its position in the different MPTs varies between a basal Somphospondyli and a sister taxon of Titanosauriformes, with its position highly influenced by the location of *Lusotitan* in each tree. Interestingly, *Europatitan* is always recovered closer to the Jurassic *Lusotitan* than to the contemporaneous *Tastavinsaurus*. To further explore the relation between the Iberian taxa, two additional constrained analyzes were performed: one enforcing the monophyly of *Tastavinsaurus* + *Europatitan*, and another enforcing the monophyly of a wider Iberian clade, formed by *Tastavinsaurus*, *Europatitan*, *Aragosaurus*, *Galvesaurus*, and *Lusotitan*.

The first constrained analysis (Fig. 19B) resulted in 20 trees of 1,113 steps. The strict consensus places the *Tastavinsaurus* + *Europatitan* clade as the sister taxa of Titanosauriformes (Brachiosauridae + Somphospondyli). Templeton’s test does not allow rejecting this topology, as it is only one step longer than the most parsimonious trees. Nevertheless, the *Tastavinsaurus* + *Europatitan* is supported by only two synapomorphies, (ch. 199, 0 → 1; ch. 213, 2 → 1). Both characters, especially ch. 199, 0 → 1 are widely distributed through Neosauropoda, with multiple reversions and convergences occurring.

The second constrained search resulted in ten trees of 1,115 steps, three steps longer than the most parsimonious trees (Fig. 19C). The resulting consensus is poorly resolved, with the Iberian clade recovered in a polytomy with *Tendaguria*, Brachiosauridae and Somphospondyli. Again, Templeton’s test failed to reject this topology at any confident level, but in this occasion the Iberian clade is not supported by any synapomorphies. The topology of this clade is also odd, with the Berriasian *Aragosaurus* as the basalmost member of the clade, and with *Europatitan* closer to *Lusotitan* and *Tastavinsaurus* closer to *Galvesaurus*.

To summarize, the current dataset fails to find evidence supporting or against the existence of an Iberian clade of basal Titanosauriformes. The general lack of support for the clades recovered in our analysis, mainly caused by the fragmentary condition of most of the specimens included, results in that many different topologies can be obtained when trees with few steps more than the MPTs are considered. This is a common problem in dinosaur phylogenetic analysis (Butler, Upchurch & Norman, 2008; McDonald, 2012) and is even more severe in sauropod datasets (Mannion et al., 2013; Upchurch, Mannion & Taylor, 2015) where the rule is that the strict consensus is very poorly resolved, recovering only a few clades with relatively good support, with Macronaria a particularly low supported clade in all the analysis. Adding new, relatively complete specimens, such as the holotype of *Europatitan* to future datasets, together with the revision of scorings of previously known specimens will help to improve our knowledge of this hotspot of sauropod evolution.

## Discussion and Conclusion

For the first time, all known material of the sauropod from the site of El Oterillo II in the Castrillo de la Reina Formation (upper Barremian–lower Aptian) of Burgos (Spain) is described with the name *E. eastwoodi*. The holotype of this new sauropod presents a series of autapomorphic characters in the posterior cervical vertebrae, the middle-posterior dorsal vertebra, the posterior dorsal ribs, and the scapula, indicating that *E. eastwoodi* is a previously undescribed taxon clearly distinct from other sauropods of the Early Cretaceous of Spain. The phylogenetic study based on the proposal by Carballido et al. (2015) allows it to be located among the somphospondylan titanosauriforms, clearly differentiated from sauropods of the other clades of Titanosauriformes such as Brachiosauridae, Euhelopodidae and Titanosauria. According to our phylogenetic hypothesis, *Europatitan* would be one of the basalmost somphospondylans, in a position close to other basal somphospondylans such as *Tendaguria* and *Sauroposeidon*.

**Figure 20:**
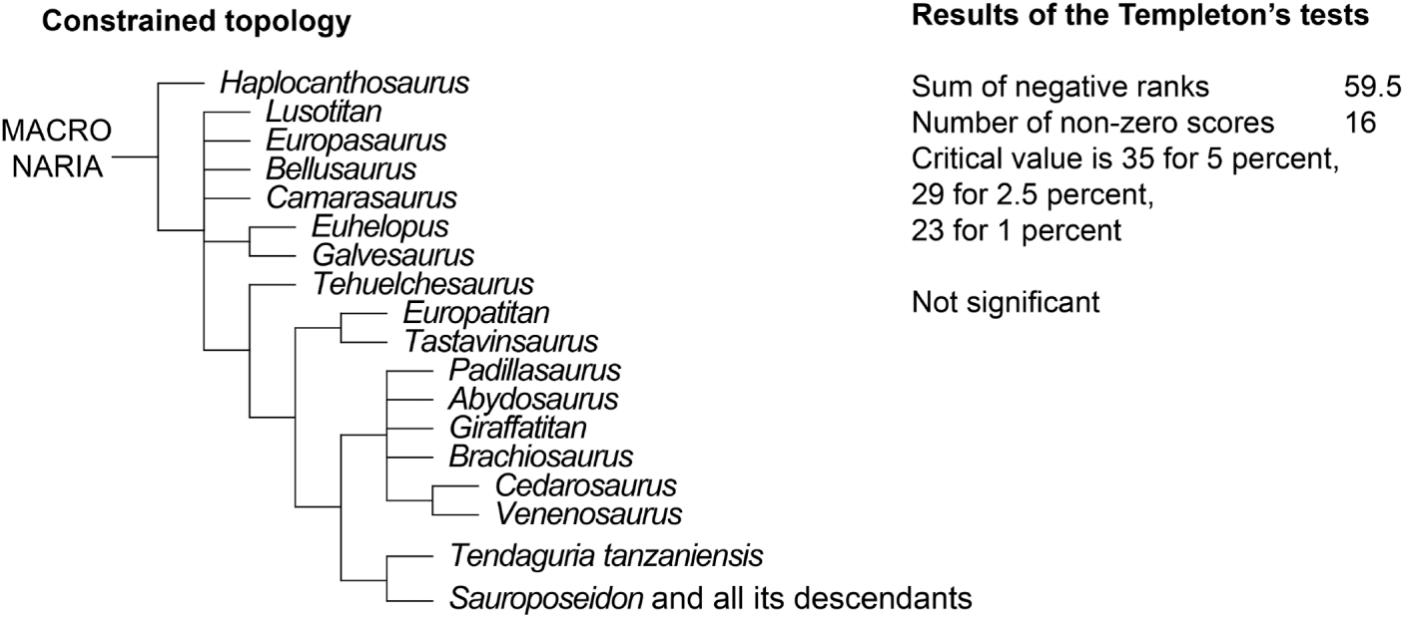
Topology of the subtree including all macronarians after enforcing the monophyly of *Tastavinsaurus* + *Europatitan*. Consensus of the 40 trees of 1,103 steps obtained after a constrained search enforcing the monophyly of the clade *Tastavinsaurus + Europatitan*, and results of Templeton’s test comparing the first most-parsimonious trees with the first constrained tree.

**Figure 21:**
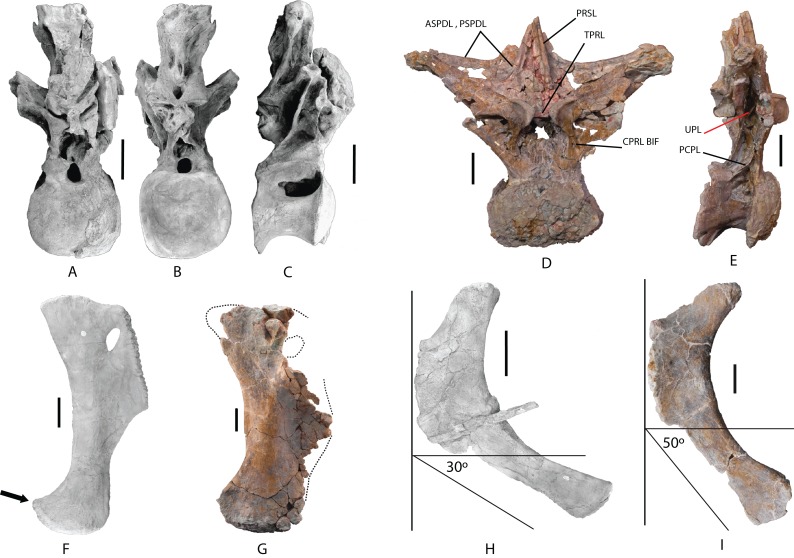
Comparison of selected anatomical characters of *Tastavinsaurus* (A, B, C, F, H) with *Europatitan* (D, E, G, I). Dorsal posterior vertebra Ars1-98 of *Tastavinsaurus* in anterior (A), posterior (B), and right lateral (C) views. *Europatitan* shows several laminae in its mid-posterior dorsal vertebra MDS-OTII,1 in anterior (D) and right lateral (I) views that do not have *Tastavinsaurus*. The pubis Ars1-16 of *Tastavinsaurus* (F, lateral view) has an anteroventral corner of the distal extremity acute (black arrow), directed anteroposteriorly. The pubis MDS-OTII,11 of *Europatitan* has this distal corner rounded (G, lateral view). In the Ischia Ars1-24 of *Tastavinsaurus* (H, medial view) the ischial ramus forms an angle of 30° respects the horizontal. In the ischium MDS-OTII,13 of *Europatitan* (I, medial view) this angle has a value of 50° (less posteroventral orientation). ASPDL, anterior spinodiapophyseal lamina; CPRL BF, bifurcated centroprezygapophyseal lamina; PCPL, posterior centroparapophyseal lamina; PRSL, prespinal lamina; PSPDL, posterior spinodiapophyseal lamina; TPRL, intraprezygapophyseal lamina; UPL, unnamed parapophyseal lamina. Scale: 10 cm.

Our analysis shows an unexpectedly distant relation between *Europatitan* and *Tastavinsaurus sanzi*, a sauropod described in a geological level of similar age in Spain, initially considered a basal somphospondylan (Canudo, Royo-Torres & Cuenca-Bescós, 2008), although its position varies with the author (D’Emic, 2012; Royo-Torres, Alcalá & Cobos, 2012; Carballido et al., 2015) between a sister group to Titanosauriformes and a basal form of somphospondylan. This discussion lies beyond the scope of the present paper, but bearing in mind the phylogenetic, geographical, and chronological proximity of *Tastavinsaurus* and *Europatitan*, it seems relevant to demonstrate clearly that they are indeed distinct taxa. In the phylogenetic proposal used in this paper, *Tastavinsaurus* is recovered as the sister taxon of Titanosauriformes. To further test the hypothesis of the sister–taxon relationship between the two Spanish taxa, a constrained search was carried out, enforcing the monophyly of the *Tastavinsaurus* + *Europatitan* clade, otherwise maintaining all the settings used in the first analysis. Only two steps more were required to satisfy this constraint, resulting in 40 trees of 1,103 steps (see topology of the area of interest in Fig. 20). The resulting consensus is similar to the consensus of the MPTs. Templeton’s test indicates that there is no significant difference between the constrained MPTs and the unconstrained topology, thus making it impossible to reject the hypothesis of a sister–taxon relationship between *Tastavinsaurus* and *Europatitan* solely based on the cladistic analysis. Nevertheless, *Europatitan* can be clearly differentiated from *Tastavinsaurus* by the bones that they share. Some of these differences are found in the dorsal vertebra and the hip bones (Fig. 21). The laminae present in *Europatitan* and their arrangement in the dorsal vertebrae are different from in *Tastavinsaurus. Europatitan* possesses anterior and posterior spdl laminae and a prsl lamina; the cprl lamina is forked at its base; and the tprl lamina links the prezygapophyses. These characters of the laminae are not present in *Tastavinsaurus*. Moreover, the spdl joins the spol, and the pcpl joins the acpl, junctions that are not present in the dorsal vertebrae of *Tastavinsaurus* (Figs. 21D and 21E). The cranioventral corner of the distal extremity of the pubis is acute in *Tastavinsaurus* (autapomorphy) and rounded in *Europatitan* (Figs. 21F and 21G). The distal extremity of the pubis of *Europatitan* is much wider than in *Tastavinsaurus*. The pubic ramus of the ischium is at an angle of 45°–50° in relation to the horizontal in *Europatitan* and 30° in *Tastavinsaurus* (Canudo, Royo-Torres & Cuenca-Bescós, 2008), Figs. 21H and 21I.

*Europatitan* provides us with new information on the initial radiation of the somphospondylans in the lower Cretaceous of Laurasia, which could have taken place in Europe. The phylogenetic proposal used in this paper separates *Europatitan* from the brachiosaurids that represent an early radiation of Titanosauriformes at the end of the Jurassic. *Europatitan* would be a representative of the Eurogondwanan fauna (Ezcurra & Agnolín, 2012), like *Demandasaurus*, the other sauropod described from the Castrillo de la Reina Formation. These authors suggest that an exchange of vertebrate faunas between Gondwana and Laurasia took place at the beginning of the Early Cretaceous, whereas in the post-Barremian period the processes of dispersal occurred independently in Laurasia and Gondwana. However, other authors have argued based on the theropod record that there was a process of dispersal between Gondwana and Laurasia at the end of the Barremian (Gasca, Canudo & Moreno-Azanza, 2014). The sauropods of the Castrillo de la Reina Formation (*Europatitan* and *Demandasaurus*), as well as the new interpretations of the sauropod *Rebbachisaurus* (Wilson & Allain, 2015), also seem to indicate that the dispersal did not break off in the Barremian.

## Supplemental Information

10.7717/peerj.3409/supp-1 Supplemental Information 1 Matrix of characters based on Carballido et al. (2015).

10.7717/peerj.3409/supp-2 Supplemental Information 2 Constrained search used to test the relation between Europatitan and Tastavinsaurus.

10.7717/peerj.3409/supp-3 Supplemental Information 3 General view of the site El Oterillo II.

10.7717/peerj.3409/supp-4 Supplemental Information 4 Detail of the articulated caudals and the dorsal vertebrae in the site.

10.7717/peerj.3409/supp-5 Supplemental Information 5 Detail of the excavation of the scapula MDS-OTII,14.

## Additional Information and Declarations

### Data Availability

The following information was supplied regarding data availability: The raw data has been supplied as Supplemental Dataset Files.

### New Species Registration

The following information was supplied regarding the registration of a newly described species: Publication LSID: urn:lsid:zoobank.org:pub:E76E9C58-CB53-4CBE-8CF5-87561A5365A1. Genus name: urn:lsid:zoobank.org:act:29532C3F-4E3F-4702-845A-2D75EF3C63B. Species name: urn:lsid:zoobank.org:act:B436CCB2-6E5C-498E-80A5-4BF271AC3175.
