# Supplementary material for: Europatitan eastwoodi, a new sauropod from the lower Cretaceous of Iberia in the initial radiation of somphospondylans in Laurasia
Source: PeerJ. 2017 Jun 27;5:e3409. doi: 10.7717/peerj.3409 (PMC5490465; doi:10.7717/peerj.3409)
Supplement: Supplemental Information 1 [file peerj-05-3409-s001.rtf]

Date S1. Matrix of characters based on Carballido et al. (2015). 

75 terminal taxa coded for 370 characters.

nstates 8 ; 
xread 'Data from Torcida et al...' 
370 75 
Plateosaurus_engelhardti 0000000000000000001000000000100000000010010?00000?0000??000000000000000??00000?0000???0000000000000?0?00?000000?00?101000?000?000000?000000000?1?0?00000000000000000??00??0000000????0?000?0???000??0?000???0?0000002?010000????0???00000000?000000?0?0???0000000000000?000000001000000000000?0000000?0000000000000?000000000000000000000000000000000???0???0010000000000010000010 
Chinshakiangosaurus_chunghoensi ????????????????????????????????????????????????????????????????????????????????0?0???00?????0?1?10???01?000??0??0??0????????????????????????????????????????????????????????0??????0?????????????????????????????????????????????????????????????????????????????????????????????????????????????????????0?????????????????????????????????????????????0????????????????????????? 
Mussaurus_patagonicus 00000000000000000010100?00?010?0000000?002???000??0?0???????????????????????????1?1???00000??0?0??010?0[01]?[01]0??00??0?10100000000???0?0??00000000?1?0?000??000000000000??00??0000000???10?000?0???000??0?0?0???0?00?0002?01000?????0???00000000?000000?0?????00000000000?0?000??000000?0[01]0000000?00?000??00000?0000000?0?0???00??000??000?0000000???00?0???0???001000??0000??1?000?10 
Antetonitrus_ingenipes ??????????????????????????????????????????????????????????????????????????????????????????????????????????????0??0?10????????0?????0??0000001121?0?00000000000000000??01??000000???????0???0???000??0?0?0???0??000002?0?000?????0????0?1010??0000??????????0000?0??0110?000??000?00?0?????????????????????00100000010001?01????????001?1?000????????????????????????0???????000??? 
Lessemsaurus_sauropoides ??????????????????????????????????????????????????????????????????????????????????????????????????????????????0010???1000000?0???000??00000010?1?0?0000000000?000000??11??000000???????????0?????????????????????????????????????????0010100?000000?0??????110010[01]001110000??000?00?0??00000?00???????00000000000001000????0?0000??00??1??000??00?????????????????0??????01000?011 
Gongxianosaurus_shibeiensis ?????????????1??????????????????????????????????????????????????????????????????????????????????????1?11?2????0??0?10????????0????????0??????????0???0???????????????????????????????????0?0???000???????????????00???01000?????0????001000??000?00?0??????1000?0?00?01?00000?0???????????????????????????111???0?????01??0???????0000?1?000000100000???1???????????0??00????????0 
Amygdalodon_patagonicus ??????????????????????????????????????????????????????????????????1???1????????????????????????????11011?20???1????1????????00????????0???0????1?????1????0??????0???????????00???0????????0????00?????????????0000???0???0??????????????????????????????????????????????????????????????????????????????????????????????????????????????????????????00?1??????????00????????????? 
Isanosaurus_attavipachi ??????????????????????????????????????????????????????????????????????????????????????????????????????????????1??0?10????????0????????0??????0?1???0????????0??????10?11??00?0???????????????????????????????????????????????????????000000??000??????????????????????????????????????????????????????????1110010001????????????????????????????????????????????????0????????????? 
Vulcanodon_karibaensis ???????????????????????????????0????????????????????????????????????????????????????????????????????????????????????????????????????????????????????????????????????????????????????1????0?0?0??00???0?100?????01???????????????00??10?????0???????????????1101?0???1110100????0???????1?????0000000?0100?101??????1000?0000?1000010?100100100?011001??????????????????????????0?0 
Tazoudasaurus_naimi ??????????????????????????????00?10?????????01?????0????????????????????????????100???0011???0?001001011?000??1000?00100000010000?010?00000110?000?0000001000011?00100111?000010??00???????0100000000001001?0?00000020020000??1100??1000000???????0?00?????1101101101?10100?100?001?0101?????00000000?10?0101010000?000?0?00010100?00?011?0?0001010?100?1????????0000000?001000100 
Shunosaurus_lii  0100100000110110001010000000??000001001??2000001101100000100011000000000??000001100?0000?101?110?1011011?201201001000100000010000100?101000110?1?1?0000000000011101[01]??11[01]?000010000010?0001010000000000100100?00?0002001?00???111010100000010??00001000010011011010010101000100000110101100000100000???00011100110001101?100?10?00111101110111?1111100101???0???000000000000000??? 
Barapasaurus_tagorei ????????????????????????????????????????????????????????????????????????????????????????????????????1?11?000??1000?0?0?0???0100????0??0000011??0?1???1010000?011100100111?000010???01100?0?0?0?00000000100??0??000?010???0?0??1?00?0110000000000000?00??????101101001110100???????????011000?0100000001000111000100?0101111??1?10???010???????1??11?0???????????????0??0?0?0000??? 
Cetiosaurus_oxoniensis ??????????????????????????????????????????????????????????????????????????????????????????????????????????????101102?000000010110100??01000110?001?0?20000100?1101000?11??0000[01]0?10??????0?02?0000000?0?0?100?000000200?000??????????00000001??0???1???0100?10?10??0???????????????????11000?0?0000??1?0?011???????100????????????????????????????????????????????000??0?0?00????? 
Patagosaurus_fariasi 1???1?00??11???????????????????0???????????????????????????????1?????????????????0???????????1???1011011?000??100100?000000010110100??00000110?001?00000011000110010?011??000010??002??0?0?????000000??1??1????0?0002002?0??????00??11000001?000?00100??????101001001110100????????????11000?010000000100011100010000001???????????101011????????????00?1???0????0000000?000000??? 
Omeisaurus       11001?00001101110010100??000???001010011010001011011?000?1???11100?0?0??00?0?00110000000?1???111?1011011010040111230000[12]000101100100?21000[23]111001100000100100?11110100111?000210010?2110100?10000000000100100?00?0002001?00???1100001100000??000?000000010011011010010101000100000011101100000100000001001111000000001011??0010100111111111111111111000?1000000000000000?000000000 
Mamenchisaurus   11101?0?001110110011100?0?00???00?010010010?010?1?111000?0???101?00??????0?0???1100???001101111001011011?1004011123[12]00020001011001010210013111001101100100?000110021001?1101021001002??0?00?11003000000100100?00?0002002?0????1100?0110?0?0?1????00?0?0????11011010011101001101?0001???1100000??000000100011100010001101?11001110?11?101?1?1111??1???00?10000000[01]00000000000000000 
Turiasaurus_riodevensis ??????????????????0???0?????????????????????????????????????????????????????????????????????????????10110001??11120100000100101?0101??11[01]1011100010??00000100011101100111100?1100100???????0???02000?0?1????????????????200?????????1?????????????????00??0?10110100????10011110000111??????????????????????????????00??11?101010?11???11???1?10?111?00?1000??????000??0?00????0?? 
Losillasaurus_giganteus ???????????????????????????????????????????????????????????????????10?????0????1??????????????1??1????????????1012010000000010110100??11110111001101000100?000111011001111100110???????????0???0200?10[01]100??00000??????1????????????????????????????????????1011010?????1??????????????01?0??010?0?00?11?0???????????????????????????????????????????????010???????00??1?????????? 
Jobaria_tiguidensis 11001?000011111?0100100?0000??10010100?101000101101110???10??11?000??00?00?0?0?1100000???????1100?01101100001?1??211?000000010010100?201010110?0[01]1?11000001000111011001111000000010021101?10100000??000100?00?100000200100????1?00101101010????01000000????110110100111010001110000111111000?0100000001100111000100?110111011111001111011111?????????00?1???000000000000?000000?00 
Haplocanthosaurus_priscus ????????????????????????????????????????????????????????????????????????????????????????????????????????????[1234]?101211?001000010010100?01111011100110110001010001100110011101001101100211011?010000000000100100?10000120010???????10??11000211?000100?00?000????????????????????????????111000?01000000011001110001001????????1????????????????????????????000??????0000?0???0?????? 
Camarasaurus     1110100000111111010000[01]0000000100101000101000101101110001101?111000000000010000110000011111111110?011011020010101212?0[01]10100101101011210011110?001011000001000110121001111000210010?2110111010000000001100100?10000020010000??[01]0[01]0101101000101001001000000011011010011101001111110011111100000110000101100111000100111011101011100111101111111111111000?1000000000000000000000000? 
Bellusaurus_sui  1?????0???11???????????????????????10???0???0??????110?????????????000????????????????????????1101011011?200??1012120001000010110100??1001111110110110000010001101110011110002101?0?2??0?1?0?100300000110?100010000???0?000?????????11[01]000010100100?00??????10110100111010????1????????11000?011000010110011101010001101?????????????????????????????00?10000?????000000?0000????? 
Galvesaurus_herreroi ??????????????????????????????????????????????????????????????????????????????????????????????????????????????1112100012??0??12001????1?011111101111100??0?0001101[12]100111100020001012110???0???01000?0?100?0???0011???0?000?????1???1???????????1?????00000?101[01]0100???????????????????????????????0101100????????????????????????????????????????????????????????0?0????00??????? 
Tehuelchesaurus_benitezii ?????????????????????????????????????????1?????????????????????????????????????????????????????????????????????????[123]?????????????????[012]10001110?001021001011000100?0100111?000201??01[123]1???1??????????????????????????????????????????110000010100100111?????1100101011110100????????????1????0011000?1011001111000001?????????????????????????????????????000????????0????00?0????? 
Europasaurus_holgeri 11101?0?001111?0010010200?????10?1?00?010?1?01011?1110000101????100000001110?0?1100???1111?1111101011011?200?01012[01]21001101110110100??100111111011021000001000110011001110100210010021?001?0?0000000000100000010001020010000??0?10??11000001010010010000????101101011110100??11??????1111101?0110000?0110?111000100?1101110101110?11?1???1??1?1??11??010000000??0?000??100000????? 
Tastavinsaurus_sanzi ??????????????????????????????????????????????????????????????????????????????????????????????????????????????????????????????????????10012?10?00????0?000100011011100?10?001211??0?211011001000100000110010001001111000000???0?1011??????????????????????????????????????????????????111101101100001011002111011?011101110???????11111110111?100111?????000????????0101???0011000 
Euhelopus_zdanskyi 01?010000111??110??????0???????00???????????0??????11?????01?1??????????????????100???1??????111??111011?2004?1112101012000010?1?001111111310110111110??0?100?11101110111100021101113??01???????????????????????????????????????????1100000?110?000?00??????10110101??????????????????1110?100110000101100111100100011011101?11?00?11101?11?1???11??000?10000?0000101????000010000 
Brachiosaurus_altithorax 12101?00??11??????????????000?10?1110?01010?01011??110??????????0?000000111??0?1?????????????1210?0?1011?201??11?222?002000111???1????10?12111001100000010100?111121001110010211???????????????0????????????????????????????????????????????????????????????10100001??????????????????????????????????????1120001000????????0????????????????????????0100000????????01??????0????? 
Giraffatitan_brancai 121010000011110101101010100000100101000100000101101110000101?111000000001110?00110000011111121210?0010110201?0111222[01]002000111110100?210012111001101020010101011[01]1210011100102110111211011?010001000000100100?1000102001000?????10??1101000?00001001100000021010010111101001111111110111110110110000101100212100100111011111?1110011110111?11?1??11?001000001111100001110001010001 
Abydosaurus_mcintoshi 121010000001?1010?1010100?0???????????0??00?01011??1100001?0?????0?0?????100????100??010????212101101011?201?01??2???????????0?????????????????????????????????????????????????1??????????00?????????????????????01?????00???????????????????????????????????????1????????????????????????????????????????2??1???0??????????????????????????????????????????111110???111????0???11 
Padillasaurus_leivaensis ??????????????????????????????????????????????????????????????????????????????????????????????????????????????????????????????????????1???2??????????0???????????1???????????2?????????????0?0001000?0010???001???1?2????????????????????????????????????????????????????????????????????????????????????????????????????????????????????????????????????000????????0111?????????? 
Lusotitan        ??????????????????????????????????????????????????????????????????????????????????????????????????????????????????????????????????????1??????????????0???????????????????????1?????????????0???00?0000010?1000100010?00?0000??0?[01]1??1???????????????????????101????0?1???00?????????????????1011??100011?0???1??????11010????1110????????????????????????1?0????????0011?0???0?0?? 
Sauroposeidon_proteles ?1??100???11??????????????????????????????????????????????????????????????????????????????????[012]?0100?011?201??1112220002000011110100??1001311121?111[12]1000010101111[01]110111?000211011121?????0???010??001100000010001020010000??0?10??110?00010000100?100???01101001011110100??1?1?11???11??0?1011?0001011002121011001110111???????????????????????????0100000??????000?010000010?1? 
Venenosaurus_dicrocei ??????????????????????????????????????????????????????????????????????????????????????????????????????????????????????????????????????????????????????????????????????????????????11???????0???0?00??00?001000100010000?000?????????11?00001?10?1???????????????????1?10100????1????????????101?0000101?00???????????????????????????????????????????????????????????111????????11 
Cedarosaurus_weiskopfe ??????????????????????????????????????????????????????????????????????????????????????????????????????????????????????????????????????100?[23]???????????????????1??????????????2?1???????????0???010??000?0?0?0010?1100000100???????????0?00010???1011??0???0210000111111??00???????????????????????????????21210110????0?????????????????????????????????????????????0?1100??0????? 
Erketu_ellisoni  ?????????????????????????????????????????????????????????????????????????????????????????????????????????????0111201?00201001101010?????????????????????????????????????????????01????????????????????????????????????????????????????????????????????1?????????????????????????????????????????????????????????????111?1101?111?0???????????????????????????????000?????????????? 
Chubutisaurus_insignis ??????????????????????????????????????????????????????????????????????????????????????????????????????????????????????????????????????1???3?10?0011?110??01?10???1?10??10?00?21???11???????010?01000?0?10??0???0001???0?000?????10??1110000111001??????????1101001111??0110??111111??????????????00010110121210110011101?????????????????1???????????????000????????0?01?01?0???10 
Tendaguria_tanzaniensis ??????????????????????????????????????????????????????????????????????????????????????????????????????????????111222000???0????00100??100??1?121?1011???0???????????001??????????????????????????????????????????????????????????????????????????????????????????????????????????????????????????????????????????????????????????????????????????????????????????????????????????? 
Wintonotitan_wattsi ??????????????????????????????????????????????????????????????????????????????????????????????????????????????????????????????????????1???31?121??1?20?????????????1??1??????21???????????001??01011???????0?010001???0?000?????10??11100001110?1????????????01000??1??0?10??111???????11????????00010?10?????????????????????????????????????????????????????????????0??????????? 
Ligabuesaururs_lenzai ??????????????????????????????????????????????????????????????????????????????????????????????[012]?0?001011?201??1?12?3?002??0???000010??1001310111?111110000101?11?111001111000211?????????????????????????????????????????????????????110000111011??????????210100011??????????????????????????????????????21210?100111011101?????????????????????????1?00000????????0????00?01[01]?10 
Phuwiangosaurus_sirindhornae ???????????????????????10?????100101????????0??????111??????????1000110?1100?0?1????????????????????1021?201[234]?101213?0?2010011100101?21[01]0131111001111101001010111121[01]011110002101???2????1?????0100?00010?1?0011001110010000??0?10??110000010001??????11????10?0000?1101110????????????1100110110001?011002121?010011111110??????????????????????????11000000???0?101101?000011??? 
Andesaurus_delgadoi ??????????????????????????????????????????????????????????????????????????????????????????????????????????????????????????????????????10?13?10?1??111??0?0101?111?11001111000211??????????001?102011000?0?00???0?01020010?0?????????????????0???????????????????????????????????????????????1011000110??00??2??????1?????????????????????????????????????000????????0101?????????? 
Mendozasaurus_neguyelap ??????????????????????????????????????????????????????????????????????????????????????????????????????????????1?1????002??0???000110??100031?121?112????0??01???????0?1????????????????????0???02000000100000010?210200110??????????1110000100011?????1?????10?100011??0?????11???????????????????????????212101?????10??1?????????????1?1???????????????????????????10110[01]?0?0??? 
Malarguesaurus_florenciae ???????????????????????????????????????????????????????????????????????????????????????????????????????????????????????????????????????????????????????????????????????????????????????????0?1?0100000010?00?000?2111000110?????????1???????????????????????????????????????????????????????????????????????2100??????????????????????????????????????????????????????01????0????? 
Argentinosaurus_hunculensis ??????????????????????????????????????????????????????????????????????????????????????????????????????????????????????????????????????100130?121?112110000001?111121101111000211????[23]?????????????????????????????????????????????????????????????????????????????????????????????????1???????????????????????????????0??????????????????????????????????000????????0????????10??? 
Epachthosaurus_sciuttoi ??????????????????????????????????????????????????????????????????????????????????????????????????????????????????????????????????0???10013110?1?1111?0000002?111211101111000211??103???????110030??001???10??10?3?010?010??????????????????0???????????????111100011?00?10???????????11?11?1?1??001?1????2121001101?101?101???????11101111111?01111?????000????????0100????0?1110 
Malawisaurus_dixeyi 1120???????1??1?????????????????????????????????????????????0???????????????0???100??????????1?10???1?21?20???101303?1021010110?0100??1101311121?1122?1200?0201?01110?11110?02110?1?????????1??0300?000100??0?101010100000????0?11??1????????????00?00110???1011011110001?0??1??111????1?????????001111100?1210???????11110????????1?????????????1??????0000?0??0?000000101?010??? 
Nemegtosaurus_mongoliensis 0020?11?0?12?10?01[01]111110?0010101101?0?0120?01010?1111??2111011?1100010??00100?1101??011??01?1210?001031?202????????????????????????????????????????????0???????????????????????????????????????????????????0????????????????????????????????????????????????????????????????????????????????????????????????????????????????????????????????????????1100???0?[01]0?????????????????? 
Rapetosaurus_krausei 00201?1?1012?1010?11?1?10?0011?00001?0?0120?01010?1111??21111???11?0100???1110?1101??011?????1210?001131?20?40101303?102101011010110??10003[01]0121?112211200002011120110111?011211????3??????????03???????00??0????3?020?21???????????1110000??????00?001?????101210011?00110????1?1????11110110110001??11?021?111111???????????????????????????????????1000000?10??000?0?1010010?00 
Isisaurus_colberti ??????????????????????????????????????????????????????????????????????????????????????????????????????????????10?301?102101010000100??1000300121?1112?1200100011100100111?0?1210?1?13110?0?????030?1001100000?101310200110????0?11?010100000?001000?00??????101111?11101??????????????110111?0110001111101???????????????????????????????????????????????000??????000??0??1??????? 
Tapuiasaurus_macedoi 002?111?0012?1010?2111200??0111011?1000?0???01011?1?1????11111???1?0000???1110??101????0??1??1210?001031020[23]??1??????0?2?0??11????????100031?121?1112?120?0000110??110111????11???11?????????????????????????????????????????????????????????????10?101???????1?????110011?????1??????????????????????????2?2???????????1???????????????????1110?111?1100???0????????????????????? 
Trigonosaurus_pricei ??????????????????????????????????????????????????????????????????????????????????????????????????????????????101103?1020?0011000100?31[01]11310121?1122[02]1200002?11121110111?0?1211?????????????????????????????????????????????????????????????????????????????????????????????????????????????????????????????????????????????????????????????????????????000??????000101?????????? 
Alamosaurus_sanjuanensis ???????????????1????????????????????????????????????????????????????????????????????????????????????1031?201??1?1303?102000?00000100??10?1[23]10121?1?12212000020110211?01111000211?1?1[23]1?0??0023103001001100000?1013101110100???0?1111111000000001111110110?1?111010011100111??1111112???????1????00?1111100212???111??111111???????1??????????????????1100000?????10100011011111?10 
Opisthocoelicaudia_skarzynskii ??????????????????????????????????????????????????????????????????????????????????????????????????????????????????????????????????????1001300121?11[12]2212001020111211101111001210??1131?0?00?2210?011001100100?101?1020002001000?111110000000?001111011110111111210111101111??1111112??11111110110001?111012121011101111111010110111111011111111011110????000????????1001111?011110 
Neuquensaurus_australis ??????????????????????????????????????????????????????????????????????????????????????????????????????????????101[23]030002??00??000100??1111300121?112211200002?111211101111001211????31?0???1?30030??001100??0?1013?021?011?1?0??????11100?0000010110111?????111210011101111????????????1111?101?0001111100212101111?1111?10101101??1110?????1????????????000??????010001?111100100 
Saltasaurus_loricatus ??????????????????????????00?????1010???020?01??????????????????0100010??1?????1??????????????????????????????1013030002001010000100??1111300121?1122112000020111111101111001211????311010?1?1?03011001100??0?10131021101101?0??11?0111000000001011011110???111210011101111???????????1111111011000111110021210111111111110??????????????????????????????000?????1010101?111101??? 
Amazonsaurus_maranhensis ??????????????????????????????????????????????????????????????????????????????????????????????????????????????????????????????????????1???111????10??0???????????0?1?0111??1???????????????0???000[01]?0?1???0?1[01]1100002?1?00??????10???????????????????????????????????????????????????????0???????????????????????????????????????????????????????????????????????????????????????? 
Zapalasaurus_bonapartei ??????????????????????????????????????????????????????????????????????????????????????????????????????????????11?211001?00?????????????????????????????????????????????????????????????????????1001000110?0110010000201100??????????????????????????????????????????????????????????????????????000?0?11?0????????????????????????????????????????????????????????0??000?????????? 
Histriasaurus_bocardeli ??????????????????????????????????????????????????????????????????????????????????????????????????????????????????????????????????????1101??????????10?010??00???00101111?01?010?????????????????????????????????????????????????????????????????????????????????????????????????????????????????????????????????????????????????????????????????????????????????????????????????? 
Comahuesaurus_windhanseni ??????????????????????????????????????????????????????????????????????????????????????????????????????????????????????????????????????11011110?0210220?1??1??0???00111111??1?01??????????0?0?201400000?10?0????100002011?0??????10??1????????????00?001?????10110111?????????????????????????01010000011?0211000100??????????????????????????????????????000????????0000?0???????? 
Rayososaurus_agrioensis ?????????????????????????????????????????????????????????????????????????????????????????????????????????????????????????????????????????????????????????????????????????????????????????????????????????????????????????????????????102111001001?????????????????????????????????????????????????????????2?1????????????????????????????????????????????????????????????????????? 
Rebbachisaurus_garasbae ??????????????????????????????????????????????????????????????????????????????????????????????????????310?????????????????????????????1101?110?02?0??01210110011100101111?111010???????1????????????????????????????????????????????11021?1001001???????????????????????????????????????????????????????????????????????????????????????????????????????0000????????0????????????? 
Cathartesaura_anaerobica ??????????????????????????????????????????????????????????????????????????????????????????????????????????????1102110011??0???000100???????????????????????????????????????????????????????????1????0111000?11?100012?1??0???????????1?21110011?1?????????????????????????????????????????????????????????2110001??????????????????????????????????????????????????0?????????????? 
Limaysaurus_tessonei ???????????1?01????1?1??0000??11??00111112001?0?10111011?20?????100101010110?101????????????????????11311213??1102110011000010000100??11011110?02101101210110011000101111?1110101?0???????[12]01[02]014000001100001111000020110011?1??10??11021110011010010011???1101001011110100????????????11???00101100001101211000?001?10??1?11???0??10001111???????????110000??????000000?0???????1 
Demandasaurus_darwini ?0?0????????????????????????????????????????????????????????????????????????????10120????????2?2?1?0??311213??1102110011000010200100??11??1?10?02????0?1?0110111100101?11??1?01011???????????20140001011001?1010100??0??00??????10??????????????????????????????????????????????????????????????111????1?1211010100?????????????????????????????????????00000????0?0?????????????? 
Nigersaurus_taqueti 00201?10101100?1??2111??0000??11??0011110??11????0111011?2??????100100010??0?1?1101200100????2301?00113112132?1102110011000010200100?211?11110?02101201111110111100101111?1100101?00???1???????140??1?1?0?0?1011?00120110011?11010??110212100000100?00??????10100101???????????????????110??0???111??????1211010100?????????1?????????????????????????11000000?00?[01]000?0?000?????? 
Suwassea_emiliae 0121???1???2?????????1??01???????00?00??12100?0??1??0?10????????0????0?0?????????1?11????????2?2???1??210????0111211000100101010???1??110?11?????1021?????????????????1?????????1100???????0????1??????????????????????????????????????0000?000????????????????2????????????????????????????????????????????????????????????????????????????????????????00000????000?????00??00?01 
Amargasaurus_cazaui ???????????2?011?????????100??10?01100011210011111??????????????0011101001?0?111????????????????????????????11110231000??1?1101001?10?01020110?1?1?1100210110?10?00100111?0000101??02??1?????0?0?0??0??????010???0?020??????????????1??0??????0?1??????????1101101001010?00????????????11001??????????????1110?0?001????????1????????????????????????????????????0000????001?00??? 
Dicraeosaurus_hansemanni 0021???1???2??01?????????100???0?011000112100111111?????1?0???10001110100010?11111011????????2311?0011210203111102310000?1?1101101?11201020110?1?1?1100010110110000100111?100010110021?1?0?01100200001110010101000002012?0?1?11?00??1100000???00100??0?????1101101001110??????????????11100001100000??001011100010011101110111110??111111?11????11????100????????0000000?000?10101 
Brachytrachelopan_messai ??????????????????????????????????????????????????????????????????????????????????????????????????????????????1?0?31???0?????????????201020110?1?1?1?00010110110000100111?1000101?????????????????????????????????????????????????????????????????????????????????????????????????????????????????????????????????????????????????????????????????????????????????000????????????? 
Apatosaurus      00201?11111201111?211100101100?0010100011201010110110010020?01?00001000000000101?1?001???????2311?00013102?331111221?00201?1102101?1131000?110?02101100001100111100100111?010010110021?111201100200001111110111000002012?0111111001011000001?0011001?00000?110120100101010011110000111111000011000001?0010111100100111011101111101111111111?1111?1110?10011?0000000000000000?00001 
Diplodocus       00201011111201111121110010110010010100011201010110110010020101100001000010100101110001101101?2311?100131020331111221?00201?0112111?1131000111??02101100001100111100100111?0101101100211111201100211101111111111210003012?0111111001011000000?1001001?0000001101101001110100???????????111000011000000?001011100010111101?1011111001111111111111?11110?1001110000000000000000?00001 
Barosaurus_lentus ????????????????????????????????????????????????????????????????????????????????????????????????????????????3?111221?00?00?1112011?11310001110?02101100001100111100100111?01001011002????0?01100211101111111111210003012?011?11?00?01???000????????1??0??????0?00?0????????????????????11??0?11?0???????10111000???11????????1???????????????????????????00???????0000000000?00?01 
Europatitan_eastwoodi ??????????????????????????????????????????????????????????????????????????????????????????????????????110200??10133??002?00?11??0100??100?21?121?101110?001?001112?110?110??????0111???????0?00020000011000?000000101?0???????0?11???1010011110110?100???????????????????????1??????????????1?110010101100??????????????????????????????????????????????1???????????0001?????????? 
Aragosaurus_ischiaticus ????????????????????????????????????????????????????????????????????????????????????????????????????????????????????????????????????????????????????????????????????????????????0??1???????0??000?00001100?0000000002100000???10100?1101000101001?0?00?????0101001001110100?1111?00???????0?001?000?1011001111111001??????????????????????????1???1?0????????????????100?00?0????? 
; 

Ccode  
   -[/1  0        -[/1  1        -[/1  2        -[/1  3        -[/1  4      
   -[/1  5        -[/1  6        -[/1  7        -[/1  8        -[/1  9      
   -[/1  10       +[/1  11       -[/1  12       -[/1  13       -[/1  14     
   -[/1  15       -[/1  16       -[/1  17       -[/1  18       -[/1  19     
   -[/1  20       -[/1  21       -[/1  22       -[/1  23       -[/1  24     
   -[/1  25       -[/1  26       -[/1  27       -[/1  28       -[/1  29     
   -[/1  30       -[/1  31       -[/1  32       -[/1  33       -[/1  34     
   -[/1  35       -[/1  36       -[/1  37       -[/1  38       -[/1  39     
   -[/1  40       -[/1  41       -[/1  42       -[/1  43       -[/1  44     
   -[/1  45       -[/1  46       -[/1  47       -[/1  48       -[/1  49     
   -[/1  50       -[/1  51       -[/1  52       -[/1  53       -[/1  54     
   -[/1  55       -[/1  56       +[/1  57       -[/1  58       -[/1  59     
   -[/1  60       -[/1  61       -[/1  62       -[/1  63       -[/1  64     
   -[/1  65       -[/1  66       -[/1  67       -[/1  68       -[/1  69     
   -[/1  70       -[/1  71       -[/1  72       -[/1  73       -[/1  74     
   -[/1  75       -[/1  76       -[/1  77       -[/1  78       -[/1  79     
   -[/1  80       -[/1  81       -[/1  82       -[/1  83       -[/1  84     
   -[/1  85       -[/1  86       -[/1  87       -[/1  88       -[/1  89     
   -[/1  90       -[/1  91       -[/1  92       -[/1  93       +[/1  94     
   +[/1  95       -[/1  96       -[/1  97       -[/1  98       -[/1  99     
   -[/1  100      -[/1  101      -[/1  102      -[/1  103      -[/1  104    
   +[/1  105      -[/1  106      +[/1  107      -[/1  108      -[/1  109    
   -[/1  110      -[/1  111      -[/1  112      -[/1  113      +[/1  114    
   +[/1  115      -[/1  116      -[/1  117      -[/1  118      +[/1  119    
   -[/1  120      -[/1  121      -[/1  122      -[/1  123      -[/1  124    
   -[/1  125      -[/1  126      -[/1  127      -[/1  128      -[/1  129    
   -[/1  130      -[/1  131      -[/1  132      -[/1  133      -[/1  134    
   -[/1  135      -[/1  136      -[/1  137      -[/1  138      -[/1  139    
   -[/1  140      -[/1  141      -[/1  142      -[/1  143      +[/1  144    
   -[/1  145      -[/1  146      -[/1  147      -[/1  148      -[/1  149    
   -[/1  150      +[/1  151      -[/1  152      -[/1  153      -[/1  154    
   -[/1  155      -[/1  156      -[/1  157      -[/1  158      -[/1  159    
   -[/1  160      -[/1  161      +[/1  162      -[/1  163      -[/1  164    
   -[/1  165      -[/1  166      -[/1  167      -[/1  168      -[/1  169    
   -[/1  170      -[/1  171      -[/1  172      -[/1  173      -[/1  174    
   -[/1  175      -[/1  176      -[/1  177      -[/1  178      -[/1  179    
   -[/1  180      -[/1  181      -[/1  182      -[/1  183      -[/1  184    
   -[/1  185      -[/1  186      -[/1  187      -[/1  188      -[/1  189    
   -[/1  190      -[/1  191      -[/1  192      -[/1  193      -[/1  194    
   -[/1  195      -[/1  196      -[/1  197      -[/1  198      -[/1  199    
   -[/1  200      -[/1  201      -[/1  202      -[/1  203      -[/1  204    
   -[/1  205      -[/1  206      -[/1  207      -[/1  208      -[/1  209    
   -[/1  210      -[/1  211      +[/1  212      -[/1  213      -[/1  214    
   +[/1  215      -[/1  216      -[/1  217      -[/1  218      -[/1  219    
   -[/1  220      -[/1  221      -[/1  222      -[/1  223      -[/1  224    
   -[/1  225      -[/1  226      -[/1  227      -[/1  228      -[/1  229    
   -[/1  230      +[/1  231      -[/1  232      +[/1  233      -[/1  234    
   -[/1  235      -[/1  236      -[/1  237      -[/1  238      -[/1  239    
   -[/1  240      -[/1  241      -[/1  242      -[/1  243      -[/1  244    
   -[/1  245      -[/1  246      -[/1  247      -[/1  248      -[/1  249    
   -[/1  250      +[/1  251      -[/1  252      -[/1  253      -[/1  254    
   +[/1  255      -[/1  256      -[/1  257      -[/1  258      -[/1  259    
   -[/1  260      -[/1  261      -[/1  262      -[/1  263      -[/1  264    
   -[/1  265      -[/1  266      -[/1  267      -[/1  268      -[/1  269    
   -[/1  270      -[/1  271      -[/1  272      -[/1  273      -[/1  274    
   -[/1  275      -[/1  276      -[/1  277      -[/1  278      -[/1  279    
   -[/1  280      -[/1  281      -[/1  282      -[/1  283      -[/1  284    
   -[/1  285      -[/1  286      -[/1  287      -[/1  288      -[/1  289    
   -[/1  290      -[/1  291      -[/1  292      -[/1  293      -[/1  294    
   -[/1  295      -[/1  296      -[/1  297      +[/1  298      -[/1  299    
   +[/1  300      -[/1  301      -[/1  302      -[/1  303      -[/1  304    
   -[/1  305      -[/1  306      -[/1  307      -[/1  308      -[/1  309    
   -[/1  310      -[/1  311      -[/1  312      -[/1  313      -[/1  314    
   -[/1  315      -[/1  316      -[/1  317      -[/1  318      -[/1  319    
   -[/1  320      -[/1  321      -[/1  322      -[/1  323      -[/1  324    
   -[/1  325      -[/1  326      -[/1  327      -[/1  328      -[/1  329    
   -[/1  330      -[/1  331      -[/1  332      -[/1  333      -[/1  334    
   -[/1  335      -[/1  336      -[/1  337      -[/1  338      -[/1  339    
   -[/1  340      -[/1  341      -[/1  342      -[/1  343      -[/1  344    
   -[/1  345      -[/1  346      -[/1  347      -[/1  348      -[/1  349    
   -[/1  350      -[/1  351      -[/1  352      -[/1  353      -[/1  354    
   -[/1  355      -[/1  356      -[/1  357      -[/1  358      -[/1  359    
   -[/1  360      -[/1  361      -[/1  362      -[/1  363      -[/1  364    
   -[/1  365      -[/1  366      -[/1  367      -[/1  368      -[/1  369    
; 

proc/;
