# Supplementary material for: Europatitan eastwoodi, a new sauropod from the lower Cretaceous of Iberia in the initial radiation of somphospondylans in Laurasia
Source: PeerJ. 2017 Jun 27;5:e3409. doi: 10.7717/peerj.3409 (PMC5490465; doi:10.7717/peerj.3409)
Supplement: Supplemental Information 2 [file peerj-05-3409-s002.doc]

**DATA S2 Constraine search used to test the relation between *Europatitan* and *Tastavinsaurus***

force

=

((*Europatitan_eastwoodi Tastavinsaurus_sanzi )Barosaurus_lentus Diplodocus Apatosaurus Brachytrachelopan_messai Dicraeosaurus_hansemanni Amargasaurus_cazaui Suwassea_emiliae Nigersaurus_taqueti Demandasaurus_darwini Limaysaurus_tessonei Cathartesaura_anaerobica Rebbachisaurus_garasbae Rayososaurus_agrioensis Comahuesaurus_windhanseni Histriasaurus_bocardeli Zapalasaurus_bonapartei Amazonsaurus_maranhensis Saltasaurus_loricatus Neuquensaurus_australis Opisthocoelicaudia_skarzynskii Alamosaurus_sanjuanensis Trigonosaurus_pricei Tapuiasaurus_macedoi Isisaurus_colberti Rapetosaurus_krausei Nemegtosaurus_mongoliensis Malawisaurus_dixeyi Epachthosaurus_sciuttoi Argentinosaurus_hunculensis Malarguesaurus_florenciae Mendozasaurus_neguyelap Andesaurus_delgadoi Phuwiangosaurus_sirindhornae Ligabuesaururs_lenzai Wintonotitan_wattsi Tendaguria_tanzaniensis Chubutisaurus_insignis Erketu_ellisoni Cedarosaurus_weiskopfe Venenosaurus_dicrocei Sauroposeidon_proteles Lusotitan Padillasaurus_leivaensis Abydosaurus_mcintoshi Giraffatitan_brancai Brachiosaurus_altithorax Euhelopus_zdanskyi Europasaurus_holgeri Tehuelchesaurus_benitezii Galvesaurus_herreroi Bellusaurus_sui Camarasaurus Haplocanthosaurus_priscus Jobaria_tiguidensis Losillasaurus_giganteus Turiasaurus_riodevensis Mamenchisaurus Omeisaurus Patagosaurus_fariasi Cetiosaurus_oxoniensis Barapasaurus_tagorei Shunosaurus_lii Tazoudasaurus_naimi Vulcanodon_karibaensis Isanosaurus_attavipachi Amygdalodon_patagonicus Gongxianosaurus_shibeiensis Lessemsaurus_sauropoides Antetonitrus_ingenipes Mussaurus_patagonicus Chinshakiangosaurus_chunghoensi Plateosaurus_engelhardti* );
