# Supplementary figures and images for: Europatitan eastwoodi, a new sauropod from the lower Cretaceous of Iberia in the initial radiation of somphospondylans in Laurasia
Source: PeerJ. 2017 Jun 27;5:e3409. doi: 10.7717/peerj.3409 (PMC5490465; doi:10.7717/peerj.3409)

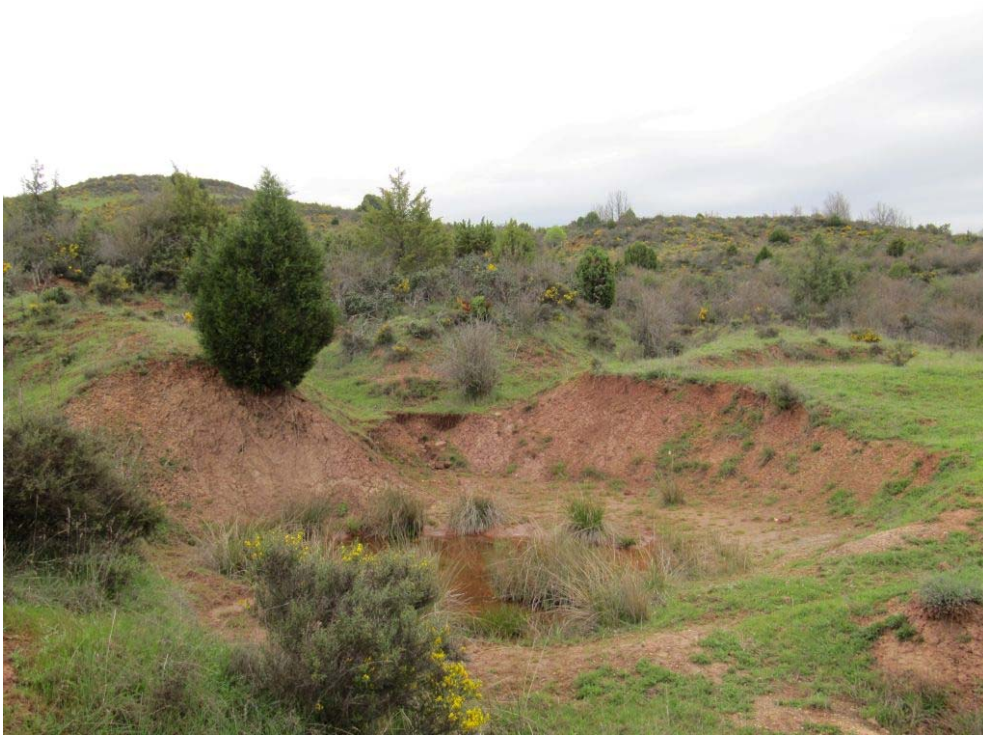

**FIG. S1** General view of the site El Oterillo II.

Supplement: Supplemental Information 3 [file peerj-05-3409-s003.pdf]

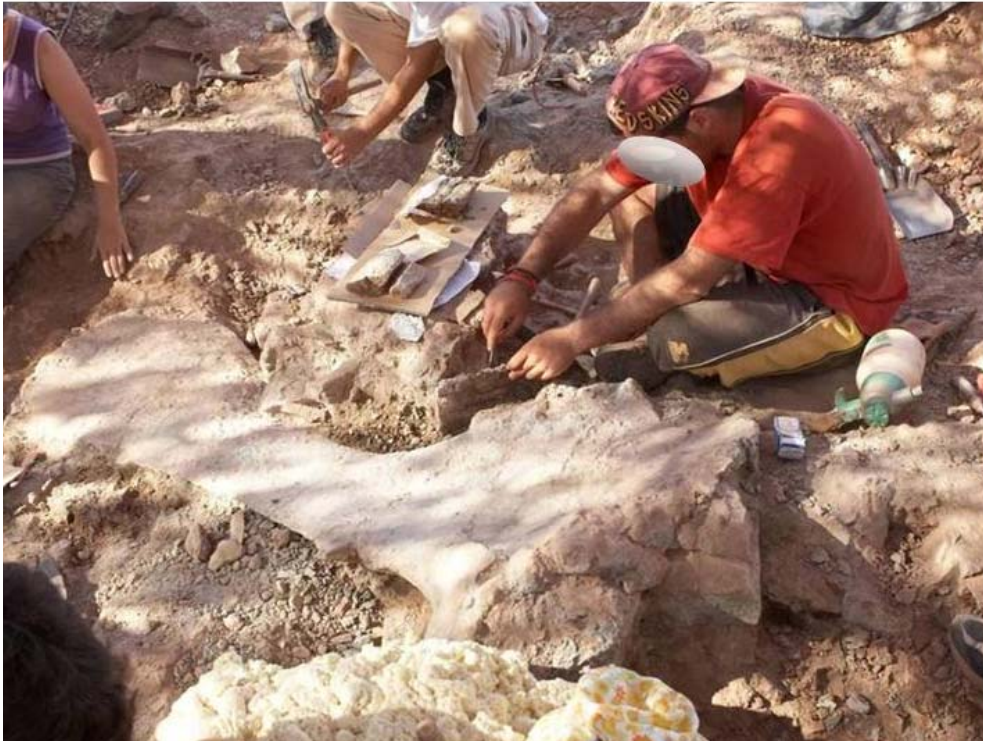

**FIG S3.** Detail of the excavation of the scapula MDS-OT,14.

Supplement: Supplemental Information 5 [file peerj-05-3409-s005.pdf]
