# Supplementary material for: Europatitan eastwoodi, a new sauropod from the lower Cretaceous of Iberia in the initial radiation of somphospondylans in Laurasia
Source: PeerJ. 2017 Jun 27;5:e3409. doi: 10.7717/peerj.3409 (PMC5490465; doi:10.7717/peerj.3409)
Supplement: Supplemental Information 4 [file peerj-05-3409-s004.pdf]

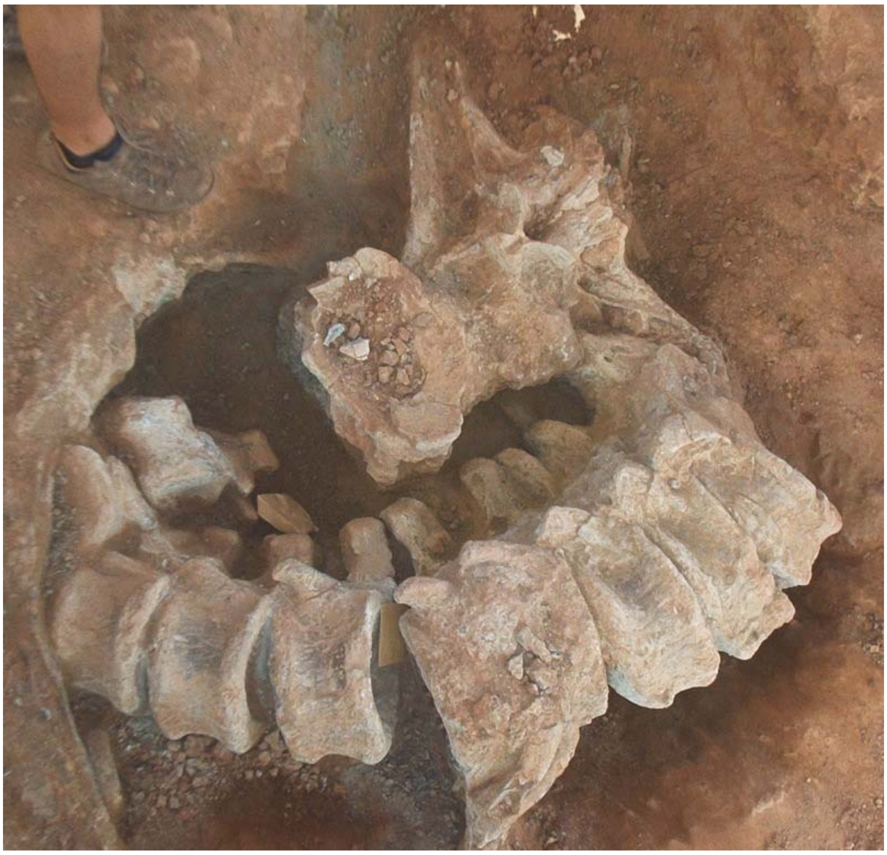

FIG. S2 Detail of the articulated caudals and the dorsal vertebra just prior to being extracted from the site.
